# A novel behavioural INTErvention to REduce Sitting Time in older adults undergoing orthopaedic surgery (INTEREST): results of a randomised-controlled feasibility study

**DOI:** 10.1007/s40520-020-01475-6

**Published:** 2020-01-23

**Authors:** Justin Avery Aunger, Colin J. Greaves, Edward T. Davis, Evans Atiah Asamane, Anna C. Whittaker, Carolyn Anne Greig

**Affiliations:** 1 School of Sport, Exercise, and Rehabilitation Sciences, University of Birmingham, Edgbaston, B15 2TT UK; 2 The Royal Orthopaedic Hospital NHS Foundation Trust, Bristol Road South, Northfield, Birmingham, B31 2AP UK; 3 Faculty of Health Sciences and Sport, University of Stirling, Stirling, FK9 4LA Scotland, UK; 4 MRC-ARUK Centre for Musculoskeletal Ageing Research, University of Birmingham, Birmingham, B15 2TT UK; 5 NIHR Birmingham Biomedical Research Centre, University Hospitals Birmingham NHS Foundation Trust, Birmingham, B15 2GW UK; 6 Health Services Management Centre, University of Birmingham, Edgbaston, B15 2TT UK; 7 School of Primary, Community, and Social Care, Keele University, Staffordshire, ST5 5BG UK

**Keywords:** Behaviour change, Intervention, Older adults, Osteoarthritis, Prehabilitation, Sedentary

## Abstract

**Background:**

Osteoarthritis is a prevalent condition in older adults that causes many patients to require a hip or knee replacement. Reducing patients’ sedentariness prior to surgery may improve physical function and post-operative outcomes.

**Methods:**

We conducted a pragmatic randomised-controlled feasibility study with 2:1 allocation into intervention or usual care groups. The intervention, based on Self-Determination Theory, involved techniques to reduce sedentary behaviour, including motivational interviewing, setting of behavioural goals, and more. The primary outcome was feasibility, assessed using mixed methods. We included exploratory measures to inform a future definitive trial, such as ActivPal3 accelerometry to measure movement, the Short Physical Performance Battery (SPPB), Basic Psychological Needs, and cardiometabolic biomarkers. Assessments were at baseline, 1-week pre-surgery, and 6-week post-surgery.

**Results:**

We recruited 35 participants aged ≥ 60 years approximately 8 weeks before hip or knee arthroplasty. Participant uptake rate was 14.2%, and retention rate 85.7%. Participants were very satisfied with the study which was found to be feasible with some modifications. Exploratory within-group comparisons found that the intervention has potential to improve SPPB by 0.71 points from baseline to pre-surgery, a clinically significant increase, and reduce sedentary time by up to 66 min d^−1^.

**Conclusion:**

In this older surgical population, it is feasible to use behavioural techniques to displace sedentary time to activity and to conduct a trial spanning the period of surgical intervention. This may improve physical function and surgical outcomes. The INTEREST intervention is now ready for evaluation in a full-scale randomised-controlled trial.

**Registration:**

This trial was registered on Clinicaltrials.gov on 13/11/2018. ID: NCT03740412.

**Electronic supplementary material:**

The online version of this article (10.1007/s40520-020-01475-6) contains supplementary material, which is available to authorized users.

## Introduction

Older adults are a growing proportion of the UK population, and ageing is associated with greater accumulation of morbidities [1, 2]. One such condition is osteoarthritis, which is characterised by a loss of cartilage with development of bone abnormalities that leads to pain and stiffness [3]. In the UK, 18.2% of adults aged 45 years and older have osteoarthritis in the knee, and 10.9% in the hip, and this prevalence rises further with age [4]. Sitting or lying while awake with an energy expenditure below 1.5 metabolic equivalents of tasks is termed sedentary behaviour [5]. Healthy older adults have been found to engage in sedentary behaviour for over 8.5 h d^−1^ when measured using accelerometry [6]. However, because osteoarthritis of the lower body causes chronic pain, it predisposes people with the condition to sit for longer than their healthy counterparts, and for longer periods without interruption [7, 8]. Older adults are also more at risk of the negative health effects of sedentary behaviour than younger counterparts, which include reduced cardiometabolic health, negative changes in body composition and physical function, and increased mortality [6]. Furthermore, sedentary behaviour has been associated with frailty independently of physical activity, indicating that sedentary behaviour may have negative physical consequences [9]. A recent harmonised meta-analysis of objectively measured physical activity and sedentary time and their effect on all-cause mortality incorporating 36,383 individuals suggests that the more sedentary one becomes, the greater the risk of mortality, although physical activity can somewhat attenuate this risk [10]. Thus, individuals who have physical ailments already and struggle to engage in physical activity, such as people with osteoarthritis, may be at even greater risk.

Individuals with severe osteoarthritis are often given a hip or knee replacement, known as an arthroplasty [11]. Arthroplasties are highly invasive and negatively impact physical function for several months after surgery [12]. The body’s ability to withstand the physiological stress of major surgery is a determining factor in the outcome, with ‘fitter’ patients (people with better physical fitness, nutritional status, and mental health) experiencing a quicker and smoother recovery [13]. In recent decades, to improve pre-surgical physical function and improve recovery rates, the concept of prehabilitation has been explored [14]. Prehabilitation involves improving a patients’ physiological reserve capacity through physical exercise, nutrition, or psychological means, or a combination thereof, enhancing their ability to recover from the surgery and to reduce complications [14].

Physical activity approaches to prehabilitation have been found to be effective. A recent systematic review of 9 RCTs encompassing 708 total patients undergoing major abdominal surgery found a significant reduction in overall mortality in the prehabilitation groups vs. control (odds ratio: 0.63, 95% CI 0.46, 0.87) [15]. It is not yet clear whether reducing sedentary behaviour by replacing it with standing and light forms of physical activity could confer a benefit to physical function, although some data suggest that this approach may have promise [16]. Two interventions to reduce sedentary behaviour in older adults have reported achieving improvements in physical function [17, 18]. One of these in 38 adults aged 60 years and older reported an improvement in Short Physical Performance Battery (SPPB) score of 0.5 points (out of a total of 12 points maximum), an increase which constitutes a noticeable positive impact in one’s mobility, after a 12-week sedentary behaviour reduction intervention [17, 19]. Likewise, correlational data suggest a statistically significant negative association between sedentary behaviour and physical function, becoming stronger the more mobility restricted the sample is [20]. More specifically, data from the osteoarthritis initiative have demonstrated that greater time spent sedentary is associated with slower gait speed independent of performance of moderate-to-vigorous physical activity [21]. Thus, it is pertinent to assess whether it is feasible to deliver an intervention to reduce sedentary behaviour in older adults with severe osteoarthritis waiting for hip or knee arthroplasties, as doing so may improve physical function, providing benefits both before and after surgery.

### Aims and objectives

The overall objectives were: (1) to assess the feasibility and acceptability of delivering a novel intervention to reduce sedentary time in a population of adults ≥ 60 years awaiting hip or knee arthroplasty; (2) assess the feasibility and acceptability of conducting the procedures required to deliver a full-scale RCT through quantitative and qualitative means; (3) estimate variance in outcome measures and the feasibility of their delivery; (4) assess intervention fidelity; (5) assess feasibility against criteria for progression to a definitive trial that would be powered to detect differences in physical function.

## Methods

This article has been written according to CONSORT 2010 extension to pilot and feasibility study guidelines for reporting randomised feasibility trials [22]. The full-study protocol has been published elsewhere [23].

### Trial design

Pragmatic, experimental feasibility study with 2:1 randomisation to experimental and control groups.

### Participants and sample size

Participants were recruited from a single site, Russells Hall Hospital, Dudley, UK, with the aid of research nurses who screened surgery lists and sent participant information sheets to eligible patients. We aimed to recruit 45 participants as a sample size of 44 would allow estimation of the retention rate of a future clinical trial with 95% confidence intervals of ± 11%, given an expected retention rate of 80%. Participants had to be (1) a man or woman aged ≥ 60 years, (2) listed for elective hip or knee surgery, (3) capable of providing informed consent as determined from their medical records, (4) able to regularly access a phone at pre-specified times, and (5) able to speak English. They were excluded if they had (1) severe neuromuscular or cognitive impairments as indicated by medical records, (2) significant comorbid disease that would constitute a risk to participation in physical activity as indicated by medical records, or (3) an unwillingness or inability to comply with the intervention. Participants were consented during the baseline data collection visit prior to any study activities (T1).

### Randomisation

Randomisation was conducted using 2:1 permuted block randomisation into intervention and usual care, respectively, by a researcher not affiliated to the project who retained allocations in confidence. This 2:1 ratio was chosen to increase our ability to assess the acceptability of the intervention in the case of under recruitment. The researcher was blinded to group allocation until the date of the visit in which the intervention was to be delivered for each participant, but it was not possible to blind the researcher thereafter.

### Interventions

#### Intervention

Participants in the intervention group were offered a behaviour change intervention based on Self-Determination Theory [24]. The intervention comprised multiple behaviour change techniques, such as social support (motivational interviewing and emotional), information about health consequences, individual feedback on current objectively measured sedentary behaviour and physical activity, goal setting (behaviour), behavioural substitution, formulation of action plans, prompts/cues, restricting the physical and social environment, review of behavioural goals and illumination of discrepancies, problem-solving, reframing, and self-monitoring of behaviour. These were delivered in two visits (visit 2 and 3, which could be combined) and took place at the participants’ homes. The intervention’s development and full details of its content and supporting materials are available in the published protocol [23].

#### Usual care

The usual care group received regular orthopaedic care except for the study assessments. This included only some physiotherapy post-operation (as did the intervention group), but no ‘training’ was provided in the usual care group prior to surgery.

### Outcomes

Data collection was taken at baseline (T1), in the week prior to surgery (T2) and 6 weeks post-surgery (T3) and occurred either at participants homes or at Russells Hall Hospital. The primary outcome for the study was feasibility, assessed with mixed methods using bespoke questionnaires given to participants, statistics regarding study processes (i.e., uptake rate, recruitment rate) and interviews with research nurses (Table 1). We assessed acceptability, practicality, adaptation, satisfaction, and safety, in line with guidance from Bowen et al. [25]. Qualitative data were collected from these sources: intervention materials (i.e., sedentary behaviour booklet, in which participants wrote their goals and recorded their adherence, available in the supplementary files of the published protocol [23]), feasibility questionnaires (which contained open questions), and a single semi-structured interview which was conducted with the primary research nurse assigned to the project. The purpose of the interview was to assess satisfaction, practicality, and ideas for adaptation of the recruitment process in INTEREST.

**Table 1:** Standard Protocol Items Recommendations for Interventional Trials (SPIRIT) diagram to show the participant schedule, including enrolment, allocation, data collection visit (T1–3), intervention visit (IV), and assessments [55]

	Study period										
	Recruitment	Baseline	Allocation	Post-allocation							Close-out
Type of contact	Pre-enrolment	Visit 1 (T1)	Allocation	Visit 2 (IV1)	Visit 3 (IV2)	PC 1	PC 2	PC 3	Visit 4 (T2)	Visit 5 (T3)	Post-study
Timepoint (weeks)	1	2	3	3	3	5	7	9	1 week prior to surgery	6 weeks post-surgery	
Study member	Research nurse	R	Third party	R	R	R	R	R	R	R	R
Enrolment											
Eligibility screening											
Informed consent		×									
Allocation			×								
Study groups											
Sedentary behaviour reduction	×	×	×	×	×	×	×	×	×	×	
Regular care	×	×	×						×	×	
Assessments											
Feasibility (study statistics)											×
Feasibility (interviews with research nurses)											×
Feasibility (questionnaire)									×	×	
Socio-demographics		×									
ActivPal measurements		×							×	×	
IPAQ-SF [29]		×							×		
MOST [27]		×							×	×	
Quality of life (QoL) (EuroQoL 5D-5L, EuroQoL-Visual-analogue scale) [56]		×							×	×	
Oxford hip and knee score(s) [38, 39]		×							×	×	
BPNS [32, 57]		×							×	×	
Activities of daily living (ADL) (Katz-ADL) [31]		×							×		
SPPB [26]		×							×	×	
SF-MNA [30]		×									
Weight		×							×	×	
Height		×									
Waist-to-hip ratio		×							×		
Cardiometabolic biomarkers		×							×		

*R* researcher, *PC* phone call, *CRP* C-reactive protein

With respect to exploratory outcomes, we collected sociodemographic data (age, gender, ethnicity, prior occupation, country of origin, educational level, pet ownership, marital status, living arrangements, alcohol intake frequency, smoking frequency, medication information, and medical history). We also assessed physical function using the SPPB [26] and included objective assessment of activity and sedentary behaviour using the ActivPal3 (PAL Technologies Ltd, Glasgow, UK) (including mean daily sedentary time, mean daily sit-to-stand transitions, mean no. of sedentary bouts ≥ 30 min per day, mean daily stepping time, mean daily standing time, and mean steps per day), and subjectively with the Measure of Older Adults’ Sedentary Time (MOST) [27]. Participants wore the ActivPal3 at all three timepoints for 3 days or greater, and the length of time worn varied from 3 to 7 days due to the amount of time available prior to surgery often being very limited. The ActivPa3 has been found to be accurate in terms of measurement of sedentariness and activity when compared with direct observation, with 100% accuracy for standing, and over 95% accuracy for stepping and sitting behaviours [28]. Physical activity was also assessed subjectively with the International Physical Activity Questionnaire Short Form [29] and nutritional status was checked at baseline using the Short Mini Nutritional Assessment (SF-MNA) [30]. We also assessed cardiometabolic biomarkers from blood samples, including albumin, high-density lipoprotein (HDL), low-density lipoprotein (LDL), cholesterol, triglycerides, vitamin D3, cortisol, transferrin, HBA1c, and c-reactive protein. Quality of Life (QoL) was assessed with Euro-QoL 5Q-5D-5L and EQ-VAS scales, and activities of daily living (ADL) with the Katz ADL scale [31]. Changes in Basic Psychological Needs, a construct within Self-Determination Theory, were assessed using the Basic Psychological Needs in General Scale (BPNS) [32]. See the published protocol and Table 1 for full details [23].

### Intervention fidelity

We assessed fidelity of treatment delivery, receipt, and treatment enactment (adherence) [33]. Treatment delivery was assessed via use of ratings of skill used by the deliverer; this included support of basic psychological needs, motivational interviewing, problem-solving, progress monitoring, and setback management. These were self-rated except for recordings of motivational interviews, which were also independently rated by an expert in motivational interviewing (CJG). Treatment receipt was assessed through independent ratings of action plans formulated by participants, according to completeness and adherence to SMART criteria, which was rated by two independent assessors. Treatment enactment was assessed via reporting of adherence to action plans (comprised of six goals and three environmental modifications) in the booklet provided as part of the intervention (available in supplementary files of the published protocol [23]).

### Data analysis and statistical methods

#### Quantitative data

We had to employ a complete case analysis approach throughout these analyses, as missing data for the final timepoint were > 50%, meaning that imputation may not have produced useful results [34]. We present the reasons for these missing data in supplementary file 6. Additionally, Bonferroni adjustments were not conducted due to the small feasibility nature of the study and potential for over-conservatism leading to type II error [35]. All tests were performed with an alpha level of 0.05 in IBM SPSS Statistics 25.0 and statistical significance is indicated in the presentation of results in Table 5; however, *p* values are not reported as this study was not powered to detect differences in these outcome measures. Therefore, statistical significance should not be interpreted as being indicative of the efficacy of the intervention.

For exploratory analysis of efficacy-related variables, we used independent group *t* tests performed on the mean differences of measures taken only at baseline and pre-surgery, and 2 × 3 (groups: intervention, usual care; time: baseline, pre-surgery, post-surgery) ANOVAs for the data taken at all timepoints.

Objective physical activity and sedentary behaviour data were calculated using the CREA algorithm in ActivPal software 8.10.8.32 with minimum upright and non-upright periods of 10 s. This algorithm automatically excludes sleeping time; however, we could not perform separate analyses for weekend vs. weekdays due to the limited number of days; it was possible to record with some individuals prior to surgery.

Within-group differences were also assessed, using paired *t* tests for variables where differences were normally distributed, and no outliers were present (as assessed using the Shapiro–Wilk test where *p* > 0.05). Within-group comparisons were performed using Wilcoxon signed-rank tests when differences were not normally distributed. If differences between timepoints were not symmetrical according to visual inspection of a histogram, then the sign rank test was used. When using the Wilcoxon signed-rank tests, we ensured that the distribution of difference scores was approximately symmetrically distributed using a histogram with a superimposed normal curve.

### Qualitative data

Both qualitative feasibility questionnaire data and adherence data from the sedentary behaviour booklet were transcribed and imported into NVivo 12 (QSR International Ply Ltd, Doncaster, Australia) for thematic analysis [36]. For qualitative data relating to adherence obtained from the sedentary behaviour booklet, an inductive approach was chosen to capture subthemes present in the participants’ experiences. These categories were then arranged into two major themes of feasibility: practicality, and satisfaction. For qualitative data obtained from feasibility questionnaires, top-level themes were based on our pre-defined feasibility criteria; however, generation of initial subthemes was based on a first-pass of the data, followed by review and refinement of these codes [37].

The interview with the research nurse was transcribed verbatim and imported into NVivo 12 for analysis. A deductive, realist form of thematic analysis was chosen, to avoid over-simplifying the data and to ensure that all aspects of the recruitment process were captured to ensure future replication and to better inform the design of a future definitive trial [37]. Deductive themes were chosen based on the main areas of feasibility by Bowen et al. [25].

Goals to reduce sedentary behaviour and environmental modifications were extracted from action plans written by participants in the sedentary behaviour booklet and imported into NVivo for analysis. These data were coded inductively into categories to create a taxonomy of behaviours that older adults are willing to engage in which can be targeted for behavioural modification. This is reported in supplementary file 8.

## Results

### Participant flow and recruitment

**Fig. 1:**
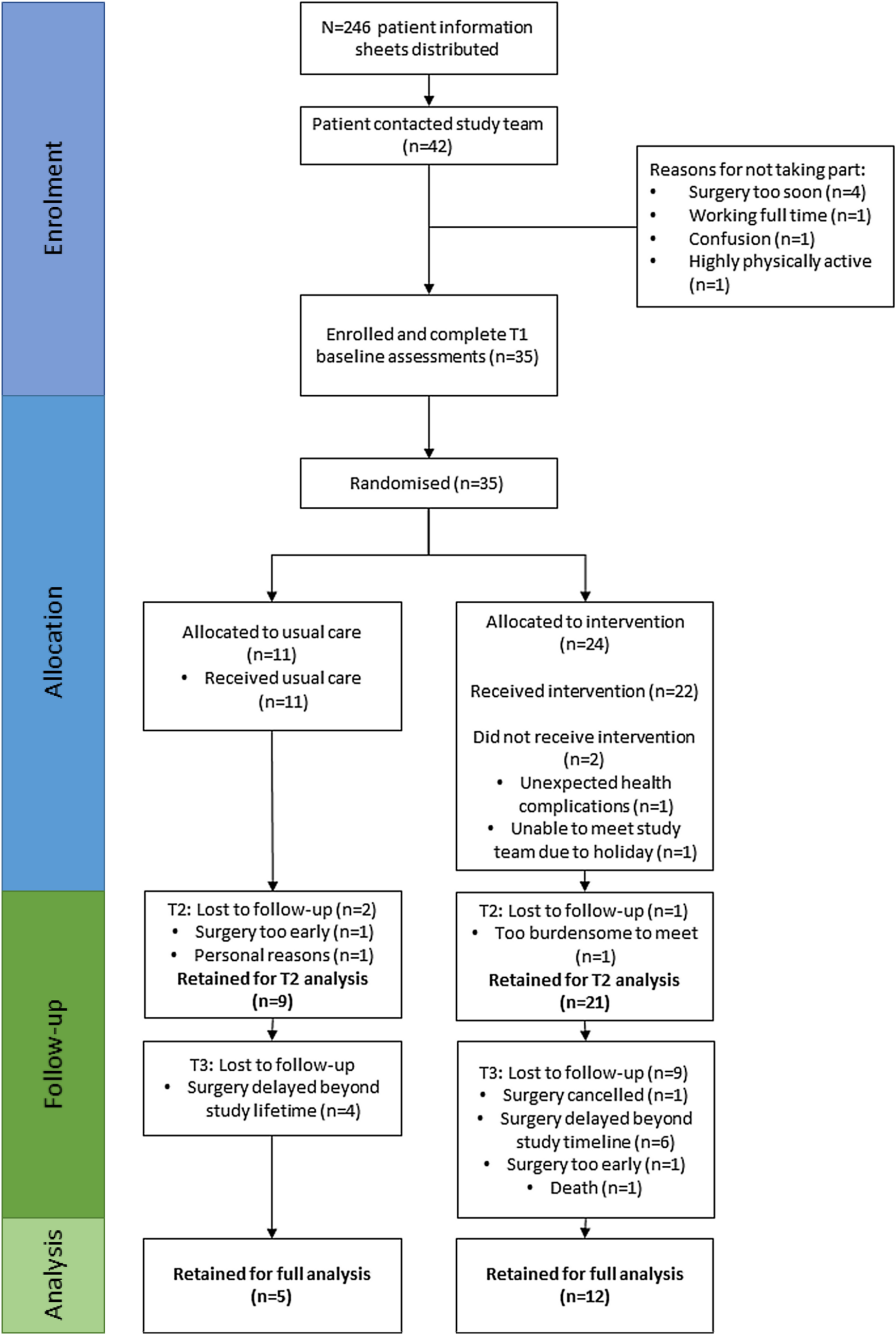
Consort 2010 participant flow diagram [33]

Recruitment began on the 29th January 2018 and ceased on the 14th January 2019 as there were no longer personnel to work on the project; study assessments continued until the 16th April 2019. Over this time period, 35 participants were recruited with a mean recruitment rate of 3.0 (SD 2.5) participants per month. As 246 participant information sheets were sent to potential participants, this yields an uptake rate of 14.2% (95% CI 10.2%, 19.4%). Figure 1 shows the flow of participants through the study. Twenty-four participants (68.6%) were allocated to the intervention group, and eleven to usual care (31.4%). A total of 112 intervention visits were conducted by the lead researcher (JAA) over a 13-month period, 111 of which occurred at participants’ homes.

The mean duration [time between first intervention delivery visit (visit 2) and pre-surgery data collection (T2)] was 59.8 (32.4) days, which was almost as intended for the planned 8-week intervention. However, the minimum number of days was 11, and the maximum was 119, which reflects the range of deviation from the actual and expected surgery dates. This may also not be reflective of the final time to surgery, as in ten cases, despite visit four (T2) having been conducted, the participant’s surgery was delayed beyond the lifetime of the study.

### Sample characteristics

Data in this section are presented as mean (SD). The characteristics of the recruited sample (and for each group) are shown in Table 2. A total of 35 participants were successfully recruited due to a lower than expected uptake rate, of whom 20 (57.1%) were women, and 18 (51.4%) were knee patients. All participants were 64 years or older with a mean age of 73.1 (5.8) years. They were almost all (97.1%) white British. The mean BMI was 30.7 (4.2) kg m^2^ and the median number of medical conditions was 3, indicating a high prevalence of multimorbidity. The sample was diverse in terms of physical function, with a Short Physical Performance Battery (SPPB) score ranging from 2 to 12, with a mean of 6.9 (2.9). Twenty-nine participants (82.8%) scored < 10, which is indicative of one or more mobility limitations and an increased risk of mortality [26]. The mean Oxford Hip/Knee score (scoring range 0–48, lower number indicates worse function) was 20.0 (8.0), which is indicative of severe arthritis that requires surgical intervention [38, 39].

**Table 2:** Baseline characteristics of sample

Variable	Intervention (*n* = 24)	Usual care (*n* = 11)	Total (*n* = 35)
Age	73.25 (5.55)	72.91 (6.54)	73.14 (5.78)
School years	11.5 (2.27)	11.36 (1.5)	11.46 (2.03)
Weight (kg)	83.89 (12.82)	83.29 (16.46)	83.70 (13.82)
Medical conditions	3.13 (1.48)	3.73 (1.74)	3.31 (1.57)
BMI (kg/m^2^)	31.00 (4.36)	30.03 (3.76)	30.70 (4.15)
Waist-to-hip ratio	0.92 (0.11)	0.92 (0.13)	0.92 (0.11)
Katz ADL (0–6)	5.62 (0.71)	5.00 (1.18)	5.43 (0.92)
MNA-SF (0–14)	12.08 (2.36)	12.27 (1.9)	12.14 (2.20)
Surgery type			
Knee	13 (54.2)	5 (45.5)	18 (51.4)
Hip	11 (45.8)	6 (54.5)	17 (48.6)
Sex			
Men	10 (41.7)	5 (45.5)	15 (42.9)
Women	14 (58.3)	6 (54.5)	20 (57.1)
Marital status			
Married	15 (62.5)	10 (90.9)	25 (71.4)
Separated	4 (16.7)	1 (9.1)	5 (14.3)
Widowed	5 (20.8)	0 (0)	5 (14.3)
Education			
Primary	0 (0)	1 (9.10)	1 (2.9)
Secondary	18 (75.00)	8 (72.7)	26 (74.3)
University	5 (20.8)	2 (18.2)	7 (20.0)
Post-graduate	1 (4.2)	0 (0)	1 (2.9)
Living status			
Alone	7 (29.2)	2 (18.2)	9 (25.7)
Not alone	17 (70.8)	9 (81.8)	26 (74.3)
Housing type			
Privately owned	21 (87.5)	10 (90.9)	31 (88.6)
Family owned	1 (4.2)	0 (0)	1 (2.9)
Public rental	2 (8.3)	0 (0)	1 (2.9)
Sheltered housing	0 (0)	1 (0)	2 (5.7)
Pets			
No pets	16 (66.7)	6 (54.5)	22 (62.9)
Dog(s)	2 (8.3)	2 (18.2)	4 (11.4)
Other pet(s)	6 (25)	3 (27.3)	9 (25.7)
Currently drinking alcohol			
Yes	13 (54.2)	5 (45.5)	19 (54.3)
No	11 (45.8)	6 (54.5)	16 (45.7)
Smoking			
Yes	1 (4.2)	0 (0)	1 (2.9)
No	23 (95.8)	11 (100)	34 (97.1)
Former smoking			
Yes	9 (37.5)	6 (54.5)	15 (42.9)
No	15 (62.5)	5 (45.5)	20 (57.1)
SPPB total points	6.92 (2.95)	7.00 (2.45)	6.94 (2.77)
BPNS autonomy	5.88 (0.94)	5.04 (1.07)	5.61 (1.04)
BPNS competence	4.63 (1.08)	4.18 (1.01)	4.49 (1.06)
BPNS relatedness	6.10 (0.92)	5.93 (0.67)	6.05 (0.85)
Oxford joint score	20.29 (7.85)	19.27 (8.71)	19.97 (8.01)
MOST total time (min.d^−1^)	485.42 (135.37)	631.04 (210.92)	531.18 (173.74)
Mean sedentary time (min.d−1)a	607.51 (125.13)	552.06 (72.21)	590.18 (113.91)
Mean upright time (min.d^−1^)^a^	327.25 (80.16)	333.8 (116.81)	329.29 (104.44)
Mean steps per day^a^	5170 (3603)	4907 (2978)	5088.25 (3374.1)
Mean sit-to-stand transitions^a^	41.05 (16.29)	35.95 (13.18)	39.45 (15.36)
Time spent in sitting bouts > 30 min (min.d−1)^a^	314.09 (165.4)	324.16 (86.88)	317.18 (144.03)
Time spent in sitting bouts > 60 min (min.d−1)^a^	173.24 (153.26)	179.20 (104.48)	175.10 (138.16)

Data are mean (SD) or *N* (%). ActivPal3 measures indicated with ^a^ are from *n* = 22 in the intervention group and *n* = 10 in the usual care group (*n* = 32 in total)

Feasibility

Feasibility—(study statistics)

Table 3 presents key study feasibility statistics.

**Table 3:** Overall feasibility statistics

Statistic	Value
Study uptake rate	14.2% (95% CI; 10.2%, 19.4%)
Recruitment rate	3.0 (2.6) participants per month
Intervention adherence	Goal data completion: 55.8% Environmental modification completion: 16.7% Mean goal adherence (where complete): 3.9 (0.7) out of 5 Mean environmental modification adherence (where complete): 4.2 (0.7) out of 5
Percentage of participants whose surgery occurred 8 or more weeks after visit 3 (IV2) (or visit 1 in usual care)	66.7%
Percentage of participants whose surgery was scheduled 4 or fewer weeks after visit 3 (or visit 1 in usual care)	16.7%
Percentage of participants with indefinitely delayed or cancelled surgery	31.4%
Retention rates	85.7% (95% CI; 69.0%, 94.6%) at T2, 48.6% (95% CI; 31.7%, 65.7%) at T3
Mean duration of intervention	Intervention: 8.5 weeks [59.8 (8.5) days] Usual Care: 16.5 weeks [115.2 (68.2) days]
Session attendance	100%

Data are mean (SD), or %

The significant rate of attrition between T2 and T3 (post-surgical follow-up) was mainly due to significant delays to the scheduled time of surgery (*n* = 10 of 13 lost between T2 and T3), rather than unwillingness of participants to attend subsequent visits.

### Intervention adherence

Out of 21 participants in the intervention group that attended the pre-surgery visit, 16 (76.2%) (T2) entered information in the adherence section in the sedentary behaviour booklet given to participants as part of the intervention [23]. Of these, there was an 88% completion rate for goal adherence. For the entries where data were provided, the overall mean of the per-participant average weekly self-reported goal adherence was 3.9 (0.7) out of a maximum of 5.

The completion rate for environmental modification adherence recording was lower, with only 52.38% of entries being complete. Where data were provided, the overall mean self-reported adherence for environment modification was 4.2 (0.7) out of 5.

#### Achievement of step targets

**Fig. 2:**
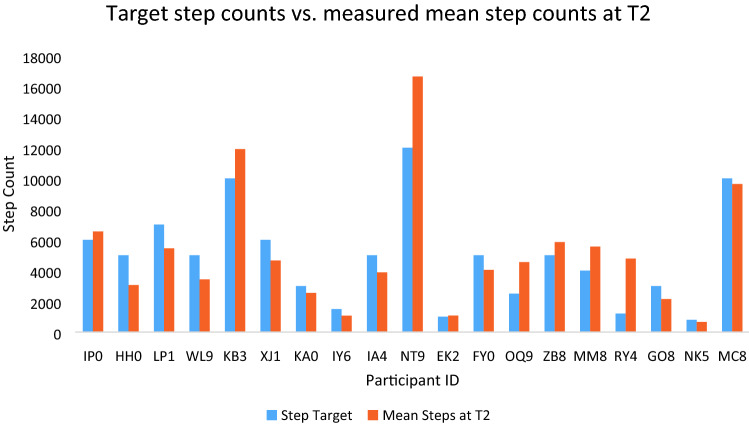
Step count targets set during intervention, versus mean daily step counts measured at pre-surgery (T2)

As part of the intervention, nineteen participants formulated goals to improve their step counts as part of their action plans and provided a follow-up ActivPal measurement at the pre-surgical timepoint (T2) (Fig. 2).

Eight (42.1%) achieved or exceeded their step targets. This indicates low adherence to the step count targets.

### Feasibility of study processes from participant perspective

#### Quantitative data

Questions were analysed according to the timepoint which they were delivered at (either T2 or T3), and by group. Full analysis is available in supplementary file 3. Thirty participants answered at T2, and seventeen at T3. At T2, participants in the intervention group reported having some problems achieving their goals in general, but no problems with the environmental modifications. They also found goals difficult to achieve physically, scoring a mean of 3.29 out of 5, but not mentally. For questions delivered to both groups, the intervention group reported the study as slightly more burdensome, but neither group found the study burdensome overall. The intervention group also reported enduring more pain, which is likely due to them attempting to increase their activity. Both groups were very satisfied with the study, reporting means of 4.4 in the intervention and 4.8 in the usual care groups (out of 5). Neither group reported feeling at risk of physical harm.

At post-surgery (T3), all intervention participants reported continuing to work on achieving goals set in the study at least “a little”, but not so much for the environmental modifications. Participants considered the study as less burdensome at this timepoint to both groups, and both groups thought that the study had a positive impact on their recovery (means of 4.5 in intervention and 4.25 in usual care group). All participants rated satisfaction of the study as a 4 or 5 out of 5.

#### Qualitative data

This paragraph presents results from the qualitative aspect of the feasibility questionnaires. Full analysis results including quotes from participants are available in supplementary file 4. In terms of acceptability, most participants focused on recording the barriers and difficulties that they encountered, which were similar to those reported from the adherence data: pain, the weather, specific activities, and problems with the pedometer. For practicality, not too many issues were faced by participants, except for further declines in physical function and other co-morbidities. For safety, the only concern was that certain activities may present additional risk for falling, particularly on wet ground (e.g., in the garden). Finally, for satisfaction and feedback, participants were mostly very satisfied with the study, reporting benefits to physical and mental health.

A total of 16 participants providing adherence data in the sedentary behaviour booklet also provided qualitative data in the “comments” sections next to the quantitative adherence rating. Full analysis is available in supplementary file 2. Participants reported that their adherence was affected mainly by pain. To avoid pain while still achieving their goals, some participants used proactive coping strategies, such as increasing painkillers and using walking sticks.

### Feasibility of recruitment—interview with research nurse (RN)

The primary roadblocks perceived by the RN that affected practicality of the recruitment strategy were problems with operating theatres and the length of participant-facing documents. Recruitment could be improved by reducing the length of these documents, increasing the recruitment period and overall study length, and by affording the RNs a better understanding of activities within study visits. For adaptation of the study, the RN did not foresee any issues with scaling to other research sites, other than that the same caveats found in this study would be likely to remain. Finally, for the category of satisfaction, the RN found that the recruitment processes in INTEREST were comprehensive, as it managed to reach all eligible patients, and the visits in participants’ homes likely improved uptake rate due to reducing burden. The full analysis is available in supplementary file 5.

### Criteria for progression to a definitive trial

Four out of five of the criteria for progression to a definitive trial were fulfilled (Table 4). Retention rate at T3 was 48.6%, lower than at T2. Most of the patients recruited in the period Nov 2018–Jan 2019 were due for surgery in Jan–Mar 2019, and only 2 out of 11 of these had surgery by Apr 2019. Only five participants who attended T3 had surgery within 4–8 weeks after the preceding visit as intended, which was 14.3% of the original total sample size, further highlighting the unpredictability of surgery scheduling.

**Table 4:** Summary of criteria for progression to a definitive trial

Criterion	Assessment	Conclusion
A minimum of 75% of patients had their surgeries within 10 days of the 4–8-week intervention window between visit 1 (usual care) or visit 3 (intervention), and pre-surgery (visit 4 or T2)	36.67% (*n* = 11) patients had the pre-surgery visit (T2) between 18 and 66 days after their preceding study visit. However, of these, only *n* = 8 had surgeries that were scheduled in the week after this visit. This constitutes only 26.67% of those retained until T2	This aspect was not feasible
Rate of uptake meets or exceeds 10%	The rate of uptake was 14.2%	This aspect was feasible
Participant retention rate exceeds 75% between baseline and pre-surgery visits	The retention rate at pre-surgery (T2) was 85.7%	This aspect was feasible
Study satisfaction must be ≥ 4/5, and risk of harm should be < 2/5, as assessed by the feasibility questionnaire given to participants	All these criteria were met at both T2 and T3. Overall mean satisfaction for both groups was 4.75. Risk of harm was assessed no higher than 1 by any participant	This aspect was feasible
The frequency of adverse events does not call into question the safety of the trial as determined by the medical expert on the study (ETD)	One adverse event occurred. This was classified as a serious adverse event, but it was not related to the study	This aspect was feasible

The unpredictability of the timing of surgery makes the prospect of fixed-time post-surgery follow-up extremely difficult and would likely negatively affect the internal validity of the trial by significantly varying intervention length or follow-up time. It is not clear whether such issues are specific to the research site or would also arise in others. Further feasibility work and internal piloting within a future multi-site trial or (pragmatically) accepting a variable “as needed” intervention duration (with refresher contacts every 2–3 months) as part of the intervention protocol may address this problem.

The overall conclusion for this study is that the study is feasible with some modifications. The study would require greater integration with hospital processes to better arrange the study around unpredictable healthcare processes. Additionally, the internal validity of the study could be improved by either:

1. mandating that the pre-surgery visit occurs at 8 weeks after baseline and post-surgery visit is cancelled if surgery does not occur within 2 weeks of this visit.
2. incorporating “top-ups” of the intervention, such that if surgery is delayed beyond a certain point, another meeting is scheduled to refresh the motivational interviewing and set more advanced or applicable goals according to the progress and feedback made by the participant.

### Exploratory outcomes and variance estimation

This section presents the statistical analyses performed on the exploratory outcomes that were collected. Baseline (T1)-to-pre-surgery (T2) results are presented separately (*n* = 30) from baseline (T1) to post-surgery (T3) (*n* = 17) results to maximise the number of participants included in the analyses.

#### Baseline (T1) to pre-surgery (T2), *n* = 30

There were no statistically significant differences present within or between groups for self-reported or objective sedentary time or physical activity measures (Table 5).

**Table 5:** Within and between-group changes from baseline (T1) to pre-surgery (T2)

Outcome	Intervention				Usual care				Mean difference in changes between groups	
	*N*	Baseline^a^	Pre-surgery^a^	Mean difference (95% CI)	*n*	Baseline^a^	Pre-surgery^a^	Mean difference (95% CI)	Mean difference (95% CI)^c^	Effect size (Klauer’s *d*)
BMI^b^	21	30.1 (3.77)	29.66 (4.01)	− 0.44 (1.65, 0.77)	8	31.07 (3.57)	30.03 (3.93)	− 1.04 (− 3.50 to 1.41)	0.60 (− 1.73 to 2.93)	0.16
Waist-to-hip ratio^b^	21	0.91 (0.10)	0.89 (0.10)	− 0.02 (− 0.05, 0.01)	8	0.88 (0.09)	0.90 (0.09)	0.02 (− 0.01, 0.04)	− 0.03 (− 0.08, 0.02)	− 0.36
SPPB total points (0–12)	21	6.95 (3.09)	7.67 (2.97)	0.71 (0.07, 1.36)	8	6.75 (2.31)	7.13 (2.70)	0.38 (− 0.88, 1.63)	0.34 (− 0.89, 1.57)	0.11
MOST Total (min.d^−1^)	21	479.66 (141.00)	463.31 (182.53)	− 16.35 (− 107.48, 74.78)	9	623.65 (186.17)	502.14 (179.70)	− 121.51 (− 251.56, 8.55)	105.16 (− 51.42, 261.73)	0.71
Sedentary time (min.d^−1^)	19	607.49 (132.62)	575.23 (84.27)	− 31.26 (− 87.42, 24.89)	6	557.28 (83.01)	562.08 (112.89)	4.80 (− 90.42, 100.02)	− 36.06 (− 144.00, 71.87)	− 0.25
Upright time (min.d^−1^)	19	322.06 (117.22)	327.89 (120.59)	5.83 (− 25.15, 36.81)	6	326.90 (105.63)	338.13 (135.87)	11.22 (− 42.37, 64.82)	− 5.40 (− 65.11, 54.32)	− 0.04
Sleeping time (min.d^−1^)	19	509.74 (112.39)	511.15 (117.91)	1.42 (− 39.52, 42.35)	6	550.78 (76.37)	495.26 (79.51)	− 55.52 (− 159.51, 48.48)	56.93 (− 28.51, 142.38)	0.53
Steps per day	19	4949.26 (3732.46)	5060.05 (3967.73)	110.79 (− 591.39, 812.97)	6	3811.17 (617.63)	3743.17 (1422.46)	− 68.00 (− 1560.67, 1424.67)	178.79 (− 1225.32, 1582.90)	0.03
Time spent in sedentary bouts > 30 (min.d^−1^)	19	314.20 (164.69)	310.68 (127.27)	− 3.52 (− 74.51, 67.46)	6	350.89 (103.67)	331.41 (107.14)	− 19.49 (− 108.22, 69.25)	15.96 (− 115.90, 147.83)	0.07
Time spent in sedentary bouts > 60 (min.d^−1^)	19	173.44 (154.82)	173.38 (85.83)	− 0.07 (− 69.14, 69.00)	6	232.00 (101.49)	199.59 (96.70)	− 32.41 (− 91.56, 26.74)	32.35 (− 93.07, 157.77)	0.11
Sit-to-stand transitions	19	41.95 (17.28)	37.95 (11.57)	− 4.00 (− 9.10, 1.10)	6	33.33 (11.72)	34.17 (9.22)	0.83 (− 6.95, 8.61)	− 4.83 (− 14.50, 4.83)	− 0.19
Oxford Hip/Knee Score (0–48)	21	20.52 (7.69)	22.67 (8.56)	2.14 (− 1.11, 5.39)	8	18.00 (9.83)	19.50 (9.46)	1.50 (− 4.38, 7.38)	0.64 (− 5.42, 6.70)	0.06
EQ-5D-5L Mobility (1–5)	21	1.86 (1.01)	2.91 (1.00)	1.05 (0.43, 1.67)**	9	1.67 (1.12)	3.22 (0.67)	1.56 (0.40, 2.72)**	− 0.51 (− 1.65, 0.64)	− 0.53
EQ-5D-5L Pain (1–5)	21	2.00 (1.30)	3.33 (0.91)	1.33 (0.58, 2.09)**	9	2.11 (1.27)	3.33 (1.00)	1.22 (− 1.0, 2.54)**	0.11 (− 1.25, 1.48)	0.09
EQ-5D-5L anxiety (1–5)	21	3.14 (1.24)	1.38 (0.59)	− 1.76 (− 2.34, − 1.19)**	9	3.33 (1.33)	2.22 (1.30)	− 1.11 (− 2.16, − 0.06)**	− 0.65 (− 1.71, 0.40)	− 0.83
EQ-VAS (0–100)	21	67.57 (21.14)	72.86 (17.36)	5.29 (− 4.97, 15.54)	9	60.56 (18.78)	59.44 (13.57)	− 1.11 (− 11.94, 9.72)	6.40 (− 10.32, 23.11)	0.48
BPNS autonomy score (0–7) ^b^	21	5.91 (0.96)	6.07 (0.74)	0.16 (− 0.26, 0.58)	9	4.89 (1.12)	5.18 (1.32)	0.29 (− 1.02, 1.60)	− 0.13 (− 1.11, 0.84)	− 0.07
BPNS competence score (0–7) ^b^	21	4.64 (1.13)	4.67 (0.96)	0.03 (− 0.51, 0.57)	9	4.13 (1.12)	4.13 (1.16)	0.00 (− 0.86, 0.86)	0.03 (− 0.93, 0.98)	0.07
BPNS relatedness score (0–7) ^b^	21	6.16 (0.90)	6.16 (0.74)	0.00 (− 0.43, 0.44)	9	5.86 (0.62)	5.71 (1.17)	− 0.15 (− 0.86, 0.56)	0.16 (− 0.62, 0.93)	0.16
Cholesterol (mmol/L) ^b^	14	4.36 (0.91)	4.29 (0.92)	− 0.07 (− 0.41, 0.27)	6	4.82 (1.42)	4.38 (0.89)	− 0.43 (− 1.46, 0.59)	0.36 (− 0.37, 1.09)	0.32
HDL (mmol/L)	14	1.39 (0.36)	1.47 (0.39)	0.07 (− 0.04, 0.19)	6	1.51 (0.37)	1.48 (0.42)	− 0.02 (− 0.23, 0.18)	0.10 (− 0.10, 0.30)	0.27
LDL (mmol/L)	14	2.31 (0.75)	2.25 (0.77)	− 0.06 (− 0.26, 0.14)	6	2.77 (1.35)	2.28 (0.79)	− 0.49 (− 1.36, 0.38)	0.43 (− 0.44, 1.30)	0.44
Triglycerides (mmol/L)^b^	14	1.46 (0.79)	1.26 (0.57)	− 0.19 (− 0.58, 0.19)	6	1.20 (0.32)	1.37 (0.44)	0.17 (− 0.12, 0.45)	− 0.36 (− 0.96, 0.24)	− 0.57
HBA1c (mmol/L)	16	39.13 (8.57)	39.44 (8.40)	0.31 (− 0.46, 1.08)	5	39.40 (1.82)	39.40 (1.14)	0.00 (− 2.91, 2.91)	0.31 (− 1.49, 2.11)	0.04
Cortisol (mmol/L)	13	334.38 (131.23)	374.62 (77.08)	40.23 (− 35.39, 115.85)	3	315.00 (22.52)	526.00 (239.90)	211.00 (− 435.21, 857.21)	− 170.77 (− 379.52, 37.98)	− 1.47
DHEAS (mmol/L)	13	1.36 (0.77)	1.30 (0.72)	− 0.06 (− 0.25, 0.13)	4	1.71 (1.18)	1.71 (1.08)	0.00 (− 0.61, 0.60)	− 0.06 (− 0.45, 0.34)	− 0.10

^a^Values are mean (SD)

^b^Shapiro–Wilk test indicated that these data were not normal in one or both groups (*p* ≤ 0.05), so within-group *p *values reported here are from Wilcoxon signed-rank or sign rank tests

^c^Calculated as “[Intervention Mean Difference T1 to T2]—[Usual Care Mean Difference T1 to T2]”

^**^p < 0.001

**Fig. 3:**
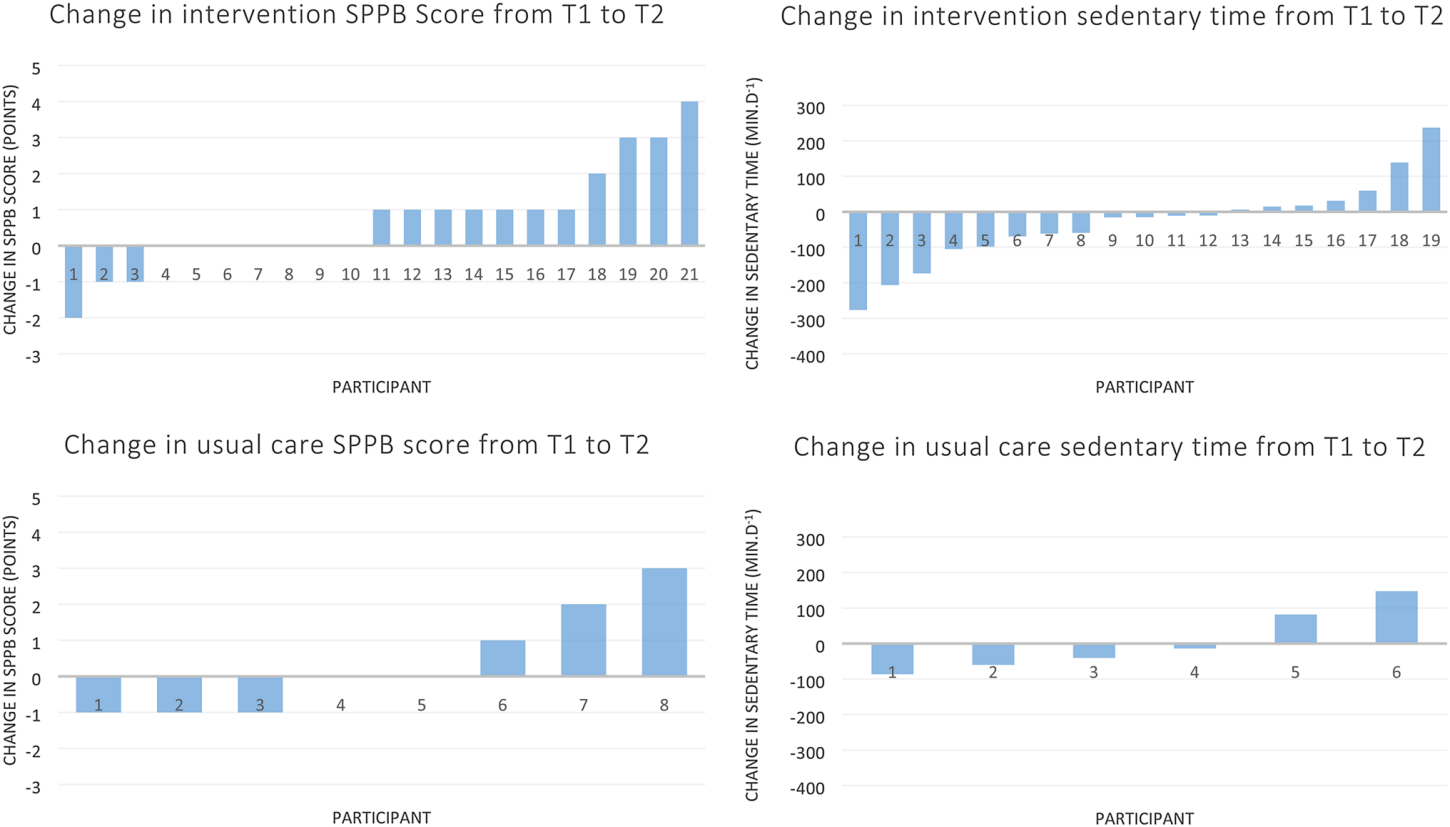
Plots of individual changes in short physical performance battery (SPPB) in each group, as well as sedentary time, from T1 to T2. For SPPB, positive change is better, and for sedentary time, negative change is preferred

A significant increase was found between baseline and pre-surgery values within the intervention group for the SPPB Total Score (mean difference 0.71 points, 95% CI; 0.07, 1.36), which is above the minimum clinically significant increase of 0.54 and is equivalent to a medium-effect size of *d* = 0.50 [40]. In the usual care group, there was a non-significant increase of 0.38 (95% CI; − 1.63, 0.88) points in SPPB score (Fig. 3).

For EQ-5D-5L scores, both intervention and usual care groups reported a statistically significant increase in the mean mobility score (indicating an increase in mobility problems) of 1.05 (95% CI; 0.43, 1.66) points, and 1.56 (95% CI; 0.40, 2.72) points, respectively, from baseline to pre-surgery. This is equivalent to moving from “no problems in walking about” to “moderate problems in walking about”. This worsening of perceived symptoms was also reflected by increases in pain score, particularly in the intervention group, wherein the mean increased by 1.33 (95% CI; 0.58, 2.09) Anxiety also decreased significantly in the intervention group by − 1.76 (95% CI; − 2.34, − 1.19) points, with a smaller, non-significant trend in the usual care group of 1.22 (95% CI; − 1.0, 2.54) points.

There were no statistically significant between-group differences from baseline to pre-surgery for any of the above tested variables. However, from baseline to pre-surgery, the intervention group had a non-significant reduction in sedentary behaviour of 31.26 min.d^−1^ (95% CI; − 87.42, 24.89), and the usual care group had a non-significant increase of 4.80 (95% CI; − 90.42, 100.02). The differences between these are equivalent to a low-to-medium-effect size of *g* = 0.32 (Hedge’s *g* for independent *T*-test).

#### Baseline (T1) to post-surgery (T3), *n* = 17.

**Fig. 4:**
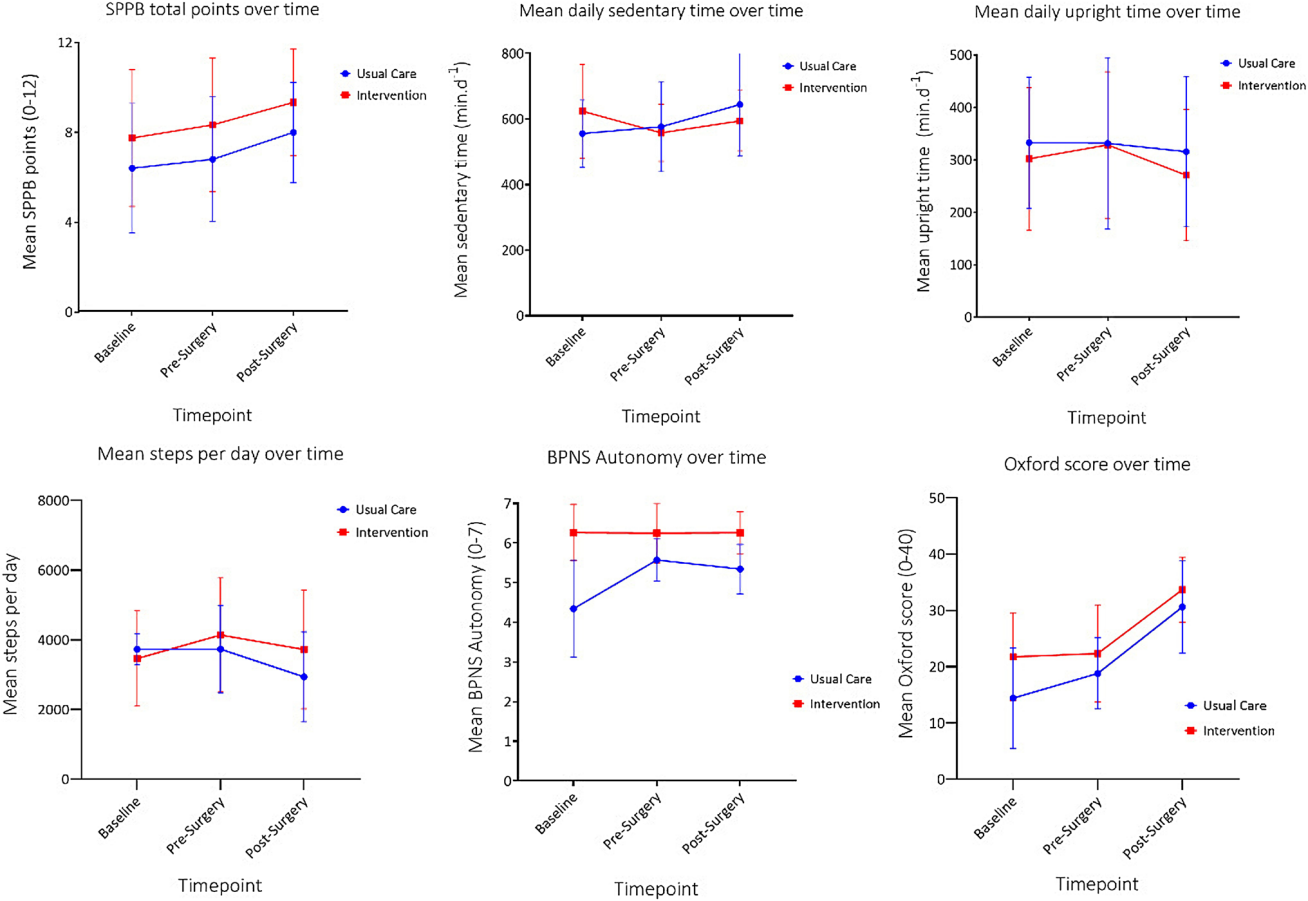
Changes over time in variables assessed within this section. Error bars are standard error. *SPPB* short physical performance battery, *BPNS* basic psychological needs in general scale

ANOVAs were performed on all outcomes (Fig. 4). However, only the variables most relevant to the logic model of the study are presented (Table 6).

**Table 6:** Change in outcomes from baseline through post-surgery in participants retained to end of study

Outcome	Group	*N*	Baseline	Pre-surgery	Post-surgery
			Mean (95% CI)	Mean (95% CI)	Mean (95% CI)
Total SPPB points (0–12)	Intervention	12	7.75 (5.90, 9.60)	8.33 (6.54, 10.13)	9.33 (7.89, 10.78)
	Usual care	5	6.4 (3.54, 9.26)	6.8 (4.02, 9.58)	8.0 (5.76, 10.24)
Sedentary time (min.d^−1^)	Intervention	8	623.85 (519.83, 727.88)	557.84 (475.36, 640.31)	594.31 (503.26, 685.36)
	Usual care	4	555.75 (408.64, 702.86)	576.48 (459.85, 693.12)	644.16 (515.40, 772.93)
Upright time (min.d^−1^)	Intervention	8	302.18 (197.54, 406.81)	328.65 (212.70, 444.61)	270.82 (167.61, 374.03)
	Usual care	4	332.91 (184.94, 480.89)	331.79 (167.81, 495.77)	315.62 (169.66, 461.57)
Steps per day (*n*)	Intervention	8	3462.00 (2541.71, 4382.29)	4138.75 (2930.16, 5347.34)	3720.13 (2466.69, 4973.56)
	Usual care	4	3730.00 (2428.52, 5031.48)	3732.50 (2023.29, 5441.71)	2935.75 (1163.13, 4708.37)
BPNS autonomy (0–7)	Intervention	12	6.26 (5.72, 6.80)	6.25 (5.82, 6.68)	6.26 (5.92, 6.60)
	Usual care	5	4.34 (3.51, 5.18)	5.57 (4.91, 6.24)	5.34 (4.82, 5.87)
Oxford (hip or knee) score (0–48)	Intervention	12	21.75 (16.74, 26.76)	22.33 (17.37, 27.30)	33.67 (29.67, 37.66)
	Usual care	5	14.40 (6.64, 22.16)	18.80 (11.11, 26.50)	30.60 (24.41, 36.79)

Within the intervention group, there was a non-significant reduction in mean sedentary time of − 66.02 (95% CI; − 180.50, 48.46) min d^−1^ in those who were retained for all three timepoints (Fig. 4, Table 6) which is equivalent to a medium-large effect size of 0.667 (Hedge’s *g*). In the intervention group, increases in upright time and steps per day were identified from baseline to pre-surgery, means of which fell slightly again after surgery, although interactions for either group or time were not statistically significant. In the usual care group, no substantial or significant within-group effects were identified. For BPNS Autonomy score, there was a statistically significant interaction between group and time, *F*(2, 30) = 0.800, *p* = 0.024, partial *η*^2^ = 0.220; however, in Fig. 4, it is apparent that Autonomy was unequal at baseline.

### Intervention fidelity

A mean score of all ratings of skills used during the intervention was computed, giving equal weighting to all skills and incorporating the independent rater for MI sessions. Overall fidelity of treatment delivery in the study was competent, with a mean of 3.1 (out of 5). According to the pre-defined progression criteria, all aspects of the study must have been delivered to the standard of 3 or above to ensure adequate standard of delivery of the study [23]. The mean of the independently rated and self-rated scores for supporting basic psychological needs during the MI session was 2.7 (out of 5), indicating that this aspect was sub-optimally delivered, which could have negatively impacted the study results. However, this independent rating only covered the motivational interview session and not the action planning portion, where this skill may have been more highly utilised.

Quality of action plans was high according to both the primary and independent rater, with all plans being rated at 5—the maximum score. A full analysis of intervention fidelity is available in supplementary file 7.

### Adverse events and safety

One serious adverse event occurred during the study: the death of a participant due to complications resulting from surgery. This was notified on the 18th of February 2019. This event was determined by the study medical expert (ETD) to be unrelated to any study procedures.

### Intervention cost

Cost per participant was calculated based on costs per participant of the present feasibility trial, comprising time and material costs. This analysis assumes that it would be delivered by an NHS staff member employed at Band 5, with hourly rate of £12.39 in the 2019/2020 pay scale. Current employer’s national insurance and pension contributions (20.68% of pay) were factored in, which were current as of July 2019. Hours required for the delivery of the study were calculated using overestimates, with 10 h for patient data collection (including travel time), giving 2.25 h for each study visit. Four and two hours were allocated for total patient-related paperwork and data entry, and for phone calls, respectively. Material costs per participant were £2.53 for a booklet, £0.10 for information sheet and other printing, and £18 for a pedometer. Overall costs came to £258.52 per intervention group participant, plus £410 one-off cost for motivational interviewing training. Including reduced usual care participant costs, the total cost for the 35 participants in the study came to £8,456. The involvement of NHS staff such as research nurses in facilitating the study and NHS overheads (indirect costs) for supporting staff set-up time, etc., were too complex to be considered in this analysis.

## Discussion

Assessment of the criteria for progression to a definitive trial found that all were met except for one related to patient surgery scheduling. As a result, the study was deemed to be feasible with some modifications. This suggests that delivering the study with a focus on the pre-surgery prehabilitative phase may be a better approach for a definitive trial, with powering based on baseline to pre-surgery changes in physical function equivalent to a 0.5 increase in SPPB score.

From the participant perspective, satisfaction with the study was very high. Participants cited benefits in both physical and mental wellbeing, and found the study practical, although difficult to achieve due to pain and health complications. Participants in the intervention group found it more burdensome and more painful, and recommended some changes to the study, such as a clearer design of study materials and more accurate pedometers for tracking of activity in this slower moving older population. The recruitment strategy was successful in identifying the target population (all participants were approached who were eligible) and home visits improved study uptake. However, an interview with healthcare staff revealed that a face-to-face strategy could have further enhanced recruitment.

There are at least ten sedentary behaviour interventions in older adults, eight of which are feasibility studies [17, 18, 41–48]. Our feasibility results are similar to results from the aforementioned studies, which have also been found to be acceptable. However, our qualitative data suggest our mobility-limited sample suffers from additional barriers to engagement in physical activity, such as pain and lower physical function. This study also suffered from slower recruitment and reduced retention rates due to the post-surgical follow-up and clinical sample. Nonetheless, this study demonstrates for the first time that it is acceptable and safe to intervene to reduce sedentariness in older adults with mobility limitations awaiting hip or knee replacement surgery.

This study also included an exploratory assessment of the impact of the intervention on several outcome variables including objectively measured sedentary behaviour at three timepoints, with the main purpose to assess the feasibility of delivering these assessments. This included, for the first time in sedentary behaviour interventions in older adults, measurement of both physical function and blood-based cardiometabolic biomarkers. Although physical function has been measured by prior interventions to reduce sedentary behaviour in older adults, measurement of blood-based biomarkers has not been performed [16, 49]. This demonstrates that it is possible for the field to begin assessing its underlying assumptions (i.e., that reducing sedentariness improves health) in both ‘healthy’ and mobility-limited older adult samples.

The exploratory analysis of ‘efficacy-related outcomes’ in this study should be interpreted with caution due to the unpowered nature of this study, and the high likelihood of type I error. However, the study found a non-significant decrease in mean daily sedentary time from baseline to pre-surgery in the intervention group, with a mean difference of -31.3 min day^−1^ and a statistically significant increase in total score SPPB score of 0.71 points over the same time period. It is worth noticing that the change in SPPB score is above the 0.54 threshold for a clinically significant difference [40]. This within-group improvement from baseline to pre-surgery is indicative of a medium-effect size (*d* = 0.50) and arose mostly due to an improvement in chair stand score. This may be due to participants in the intervention group forming goals to perform many chair rises throughout the day; however, no mean increase in objectively measured sit-to-stand transitions was found. Either participants did not perform these goals, or it is possible that the ActivPal3 device used was not able to pick up chair rises performed rapidly in succession. Since the usual care group also enjoyed a small increase in SPPB score, it indicates that there may be a ‘practice effect’ occurring as participants in both groups know that they will have to repeat the task on a later date and become more familiar with the methodology. Similar findings were identified in a prior intervention to reduce sedentary behaviour in older adults, which found an improvement of 0.53 points in SPPB (*p* = 0.046), also arising from improvements in chair rise test performance, despite no corresponding decrease in sedentary behaviour [49]. The authors posited that the specificity principle, which refers to an increase in function during specific frequently performed movement patterns, may be the underlying mechanism. However, we would suggest that the effect of practice cannot be ruled out and this impact should not be attributed to the intervention.

This study is also the first to incorporate a follow-up after a sedentary behaviour reduction in older adults; in this case, occurring 6 weeks post-surgery. An analysis of eight individuals who completed the intervention found that there was a non-significant mean reduction in sedentary time of 66 min day^−1^ from baseline to pre-surgery. After surgery, sedentary time increased by 36.47 min day^−1^ in the intervention group. Over the same three timepoints, the usual care group (comprising *n* = 4 individuals) underwent a non-statistically significant increase in mean sedentary time at each follow-up timepoint, equal to 88.41 min day^−1^ from baseline to post-surgery. Due to the very small sample size and lack of statistical significance, it is impossible to attribute this effect to the intervention. However, such a magnitude of reduction is consistent with, and slightly greater than, other studies in healthy older adults [16, 43]. Changes in cardiometabolic biomarkers were not found, either within or between groups in response to the intervention, which is in line with findings of prior RCTs and cross-sectional studies, as there is a multitude of factors that can affect these biomarkers, such as diet and medications, and it is impossible to take these all into account [50].

Statistically significant changes were also present within the intervention and usual care groups for EQ-5D-5L mobility score (which indicated worsening mobility), and for pain and anxiety in the intervention group only. However, these changes in both pain and anxiety are difficult to attribute to the intervention, as anxiety may be lowered by the imminence of surgery (whereas at baseline, surgery was far in the future with more uncertainty), and increases in pain may be attributable to progression of osteoarthritis rather than the intervention itself. This seems likely as the usual care group also trended towards a pain increase of a similar magnitude. Small-to-large-effect sizes were also identified in favour of the intervention in key variables such as sedentary time (*d* = 0.25), waist-to-hip ratio (*d* = − 0.36), EQ-VAS (*d* = 0.48), self-rated anxiety (*d* = − 0.83), and cortisol (*d* = − 1.47) from baseline (T1) to pre-surgery (T2); however, as effects were also found which favoured the usual care group, it is difficult to place much confidence in these findings. A trial with more statistical power is required to draw more informed conclusions from these outcomes.

Finally, with respect to prehabilitation, a recent review recommends that prehabilitation programmes prior to surgery should be more personalised and include psychological support, as these are lacking in existing prehabilitation programmes [51]. Since this study may have potential to improve physical function, a behavioural change approach, such as the one used here, could deliver a more personalised and similarly effective form of prehabilitation, as each participant was working to achieve their own action plans which were devised in collaboration with the researcher. Such an approach to prehabilitation may also provide other benefits such as reduced resource requirements and more sustained and self-motivated behavioural change in comparison with traditional physiotherapy programmes.

### Recommendations for a definitive trial

**Fig. 5:**
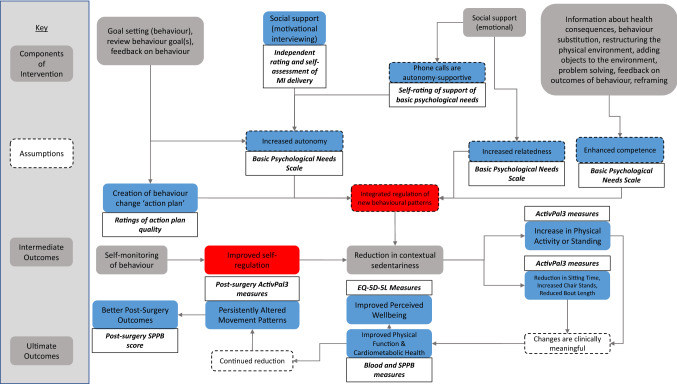
INTEREST logic model with areas assessed by an outcome measure indicated in blue, and those unassessed indicated in red. Bold, italic text indicates the outcome measure used to measure the attached outcome or assumption

Based on the findings of this feasibility study, we have a number of recommendations for design of a follow-up trial based on this study. First, we would recommend further inclusion of measures to better assess all aspects of the study logic model (Fig. 5), such as a way to measure behavioural regulation specific to sedentary behaviour—which does not yet exist. Second, further qualitative interviews with participants should be incorporated to garner additional details about which aspects of the intervention they found most useful. In addition to a 6-week follow-up, a 12-week follow-up should also be incorporated, as that is the point at which most individuals reach their post-surgery physical function peak [52]. This would allow for calculation of recovery trajectory (Δ recovery) in those in the intervention group vs. usual care. Furthermore, better integration of study procedures into healthcare practices would aid substantially in delivering aspects such as the blood biomarkers, which could be taken by research nurses around clinical timepoints (i.e., screening, surgery, and follow-ups). With respect to timing of assessments around surgery delays, to increase internal validity, the pre-surgery measurements could either be taken at a specific cut-off point for all participants, or they could keep waiting for their surgery with provision of ‘top-ups’ of motivational interviewing and revised action plans at set intervals. Additionally, access to and comparison with “usual care” data both financially and in terms of individual recovery would greatly aid interpretation of the study results. The findings of this study warrant an adequately powered follow-up trial, and as such, we performed a calculation using standard deviations obtained from this study to detect a 0.54 point change between groups in SPPB score from baseline to pre-surgery with 90% power and 10% drop-out. Although we have used effect sizes determined to be significant elsewhere in the literature, it is worth noting that using standard deviations from pilot studies for sample size calculations may be misleading due to high potential for sampling bias [53]. The calculation informed us that 250 individuals would need to be recruited, or 125 per group. This would necessitate a multi-site design and much greater resource to deliver alongside the other elements discussed above.

## Strengths and limitations

Strengths of this study include the randomised design, which is more robust than a simple pre–post-design, and the first time such an intervention has been delivered as a form of prehabilitation. It is also the first time an intervention to reduce sedentary behaviour in older adults has included cardiometabolic biomarkers as an outcome measure. Furthermore, the study used both objective assessment of sedentariness and physical activity with ActivPal3 inclinometers, and subjective assessment with the MOST, which together provides both accurate measurement of sedentariness and captures contexts for the behaviour. The inclusion of a secondary follow-up after the end of the intervention, although affected by surgery and being relatively short at only 6 weeks, also allowed assessment of the short-term persistence of any behaviour change over the post-surgical period. The wide range of mixed-method feasibility data, including interviews, questionnaires, and data recorded by the participants provided a comprehensive assessment of feasibility using pre-defined progression criteria. The study also had a strong theoretical design, including assessment of the basic psychological needs within self-determination theory [16]. The inclusion of the fidelity assessment was a further strength of this study and is a first in interventions to reduce sedentary behaviour in older adults. Although the sample was small and lacking in diversity, it was well balanced for sex and surgery type, and was reflective of the elevated degree of morbidity in this population.

This study had several limitations. First, the variable intervention length due to reliance on unpredictable surgery scheduling may have led to lack of comparability both between groups, and within groups, negatively affecting internal validity, but this is an unavoidable aspect of intervening in this clinical population. This could be avoided in future studies by mandating a cut-off point at which the post-intervention assessments must occur. Second, the assessment of intervention fidelity was (in part) self-rated and lacked robustness due to a lack of personnel to enable an independent process evaluation. The assessment of the delivery of the support of the basic psychological needs skill indicated that there was scope for improvement with the delivery of this element of the intervention. Thirdly, the small sample size, particularly at post-surgery, may have impacted the assessment of feasibility of the study, by potentially not supporting data saturation in the qualitative data analysis. This could be improved with a better-resourced study. Finally, although testing and refining the theoretical basis of the study and/or assessing its logic model was not an aim of the present feasibility study, a follow-up trial should consider the theoretical framework more heavily in the design and consider performing mediation analyses to identify what aspects of the intervention predict positive outcomes.

A lack of statistical power likely also limits the conclusions that can be drawn from the analysis of exploratory outcomes. However, as this was a feasibility study, it was not powered to detect these differences. Additionally, the study was conducted over a 13-month period, and thus, seasonality may have affected physical activity and sedentary behaviour measurements for some participants. The relative homogeneity of the sample is also not reflective of the local (Birmingham, UK) population, which is much more ethnically diverse, so the generalisability of these findings may be limited. In addition, the study was carried out by one researcher, who was not blinded to group allocation at assessment points, which may have introduced measurement bias. Furthermore, the single-site nature of this study means that findings may not be generalisable to other research sites. Including both hip and knee patients may have resulted in differing recovery trajectories, which makes interpreting results in a single analysis more difficult, and multiple clinicians were performing the surgeries, which could have further affected post-surgery recovery. Nonetheless, it was necessary to include all patients from as many clinicians as possible to ensure that adequate numbers of participants could be reached within a reasonable timeframe for the study.

## Conclusion

The INTEREST feasibility study was found to be feasible with some modifications. The primary difficulty was highly unpredictable patient surgery scheduling, affecting feasibility of follow-up and scheduling of the pre-surgery visit. This could be alleviated with small changes to the study design or better integration with healthcare services. Exploratory analysis of outcomes, although not powered to allow definitive conclusions, suggested that this novel, theory-informed intervention may have potential to reduce sedentary time and confer a prehabilitative effect by increasing pre-operative physical function to a clinically meaningful degree. Qualitative feedback from participants suggested that the pain and fatigue associated with osteoarthritis were key barriers affecting goal attainment; however, participants also reported gaining physical and mental benefits from the intervention. Given that such an intervention is acceptable and safe in patients undergoing hip and knee arthroplasty, a full-scale trial with adequate statistical power to detect improvements in physical function in older people awaiting joint surgery is warranted.

## Electronic supplementary material

Below is the link to the electronic supplementary material.

Supplementary file1 (PDF 722 kb)

Supplementary file2 (PDF 898 kb)

Supplementary file3 (PDF 833 kb)

Supplementary file4 (PDF 802 kb)

Supplementary file5 (PDF 998 kb)

Supplementary file6 (PDF 473 kb)

Supplementary file7 (PDF 638 kb)

Supplementary file8 (PDF 479 kb)
