# Supplementary material for: A novel behavioural INTErvention to REduce Sitting Time in older adults undergoing orthopaedic surgery (INTEREST): results of a randomised-controlled feasibility study
Source: Aging Clin Exp Res. 2020 Jan 23;32(12):2565–85. doi: 10.1007/s40520-020-01475-6 (PMC7680324; doi:10.1007/s40520-020-01475-6)
Supplement: Supplementary file 1 — Supplementary file1 (PDF 722 kb) [file 40520_2020_1475_MOESM1_ESM.pdf]

Assessment of Study Fidelity

Types of fidelity assessment in INTEREST

Assessment of study fidelity was also essential to both understanding the feasibility of the study, in terms of whether any aspects of the study are presenting issues in terms of delivery and was also important when assessing the feasibility of the efficacy measures used in the study. The National Institute of Health Behaviour Change Consortium outlines several aspects of fidelity that can be assessed, such as treatment fidelity, treatment receipt, and treatment enactment (Bellg *et al.*, 2004). Treatment fidelity involves ensuring quality of the methodological practises used within the study, treatment receipt is whether participants understand and have taken into account the aspects of the intervention delivered to them, and treatment enactment refers to how well participants are utilising learned treatment techniques or skills in their daily lives (Bellg *et al.*, 2004). INTEREST aimed to measure all these constructs to some extent, but mostly focused on treatment fidelity. By measuring treatment fidelity, it is possible to gain additional insight into mostly the internal validity of the study, and to have a greater understanding of why expected outcomes may or may not have occurred in the actual delivery of the study components. If an intervention does not measure treatment fidelity and has negative results, it may be inaccurately attributed to the study's methodology, whereas the study may have actually been improperly delivered.

Fidelity assessment methodology

The INTEREST treatment fidelity measure for components delivered by a facilitator uses a six-point scale measuring adult skill acquisition to reflect the competence level of intervention component delivery (Dreyfus, 2004). This scale ranges from 0, indicating an absence of skill, up to 5, which would be considered an 'expert' level of delivery (table 4).

Table 4. The generic (non-applied) 6-point score of adult skill acquisition (Dreyfus, 2004).

Competence Level	Scoring	Description
Absence	0	Does not resemble the skill attempting to be used.
Novice	1	Minimal use of the skill and /or inappropriate performance of the skill.
Advanced Beginner	2	Evidence of competence, facilitator begins to understand the context involved, but numerous problems and inconsistencies. Detached involvement.
Competent	3	Competent, aware of and able to cope with different contexts and situations, but with minor problems. More engaged involvement.
Proficient	4	Minimal problems or inconsistencies. Able to adapt appropriately to a variety of contexts and situations. Involved understanding.
Expert	5	Highly appropriate and proficient with no problems. Able to make more subtle refined discriminations between contexts and able to adjust accordingly. Highly involved understanding.

At any time, the deliverer may encounter difficulties, for example due to resistance from the participant, etc. However, in these cases the assessor should have still focused on rating the delivery of the facilitator, whatever the response of the client may be.

Performance rating process

Separate documents were created for the rating of individual sessions in the intervention (visit 2/3, phone calls, and action plan rating). Using these forms, the initial assessment should have considered whether the key features of the skill are present (e.g. for motivational interviewing, does the facilitator use OARS, etc.). Secondly, the assessor should assess whether these skills are delivered in the appropriate contexts rather than merely being present. If a facilitator utilised all the required skills in the appropriate contexts (i.e. doesn't miss many opportunities nor misuses opportunities), then they would be rated among the top competencies. INTEREST fidelity assessment covers motivational interviewing, problem solving, progress monitoring, setback managing, and action planning skills.

Performance standards

The intervention would be considered as having been delivered to a good standard if the mean competence of delivery was at level 3 or greater across all items, and if action plans scored a 3 or above.

Item 1: Motivational interviewing

Key features

The Motivational Interview (MI) should have been delivered in a client-centred manner, encouraging the patient to be the main driver of behaviour change in an *autonomous* fashion. The instructor should not have been dictatorial, judgemental, or disrespectful. Rather, they should have *supported the patient* and provide them with resources and *encouragement* with which they could realise their own capacity for change. The interaction should have demonstrated *a genuine sense of empathy and warmth* from the facilitator and should have been *individually tailored* to the patient's specific needs and should have *adapted* in response to new information. The patient should have been talking for *more than half of the time*. The facilitator could have shared their own experiences and provided *information/expertise*, but only in a manner that asks *permission*, and was *open-ended* and not dictatorial. MI techniques were also be used in the phone calls where appropriate, and the below skill table (Table 5) may also have been used to aid in skill rating after the phone calls where required. Review of the MI delivery should aid in assessing both treatment fidelity and treatment.

Intervention techniques

1. In the initial phases of the MI (engaging, focusing), OARS (open questions, affirmation, reflective listening, summaries) should be used throughout to guide the discussion.
2. Reflective listening at minimum consists of simple reflections that repeat or rephrase elements of what the patient said, but a more skilled facilitator will use complex reflections that exaggerate/amplify the reflections, reframe, emphasise personal autonomy, or reinforce key theoretical components of the intervention /logic model (e.g. highlighting participant experiences of relatedness or their growing sense of competence).
3. Change talk should have been elicited from the patient, and the deliverer should have switched to EARS (elaborating, affirming, reflecting, and summarising).
4. Summaries should have been used throughout that reinforce/affirm/praise patient effort.
5. The responses of the facilitator should have exhibited specific tailoring to the responses of the patient and there should be evidence of a collaborative relationship. There should be no evidence of arguing, disagreements, judgment, blame, or persuasion.

Table 5: MI checklist

Place an X in the box for the highest competency level for which the facilitator fulfils all criteria.

Score	Proficiency	Description of characteristics
	0 – Absence of skill	An overly directing, practitioner-led, or dictatorial style of interaction without any evidence of change talk.
	1 – Novice	Little patient involvement. Minimal evidence of use of MI techniques. The facilitator talks for most of the session.
	2 – Advanced beginner	Some evidence of MI technique usage. Facilitator dominates the discussion. Numerous problems or inconsistencies. Detached involvement.
	3 – Competent	Appropriate use of basic MI techniques (OARS) and summaries. Change talk becomes evident. Evidence of a collaborative relationship. Difficulties in content or method of delivery.
	4 – Proficient	Exclusive use of client-centred delivery style. Participant and facilitator have a collaborative relationship. Little difficulty and few missed opportunities to use MI techniques. Some use of advanced MI skills, such as complex reflections, and summaries are delivered where appropriate, in a manner that furthers the discussion.
	5 – Expert	Highly proficient use of a wide range of advanced MI techniques, e.g. complex, strategic reflections, summaries, etc. No opportunities missed or evidence of problems. Smoothly transitions with new information and uses it in the MI techniques.
	N/A	Not relevant to this activity.

Item 2: Phone call delivery

Key features

The phone calls in INTEREST served the following purposes:

1. A motivational tool if the patient shows signs of reverting to a less motivated or more ambivalent state. This is on a case-by-case basis (where required).
2. To query the patient about goal adherence and offer opportunities for problem-solving collaboratively with the patient if any goals/environmental modifications are deemed as unachievable. This should result in changes to the goals to make them achievable (where required).
3. To monitor progress.
4. To manage setbacks (where required).

Given that many techniques could have been used in the phone calls, assessment of fidelity required a number of techniques to be assessed, including MI techniques (item 1) and problem-solving skills (item 2a), progress monitoring skills (item 2b), and setback management skills (item 2c).

Intervention techniques

The facilitator should have appropriately identified in which phone calls MI techniques are required and should have used problem solving skills as appropriate to work with the patient on a solution any problems that they have identified, monitor progress, and help manage setbacks (where needed).

Due to the private nature of these calls, the practitioner engaged in a self-rating exercise soon after the call is completed (Table 6). Changes to participant goals that emerged from discussion were recorded in Table 7 on the relevant document.

Item 2a: Problem-solving

Intervention techniques

Reframing should have been used in cases where there's a setback to focus on the opportunity to use it as a learning experience and help support the patient. Goals should have been revised and reformulated where necessary as part of problem solving and progress monitoring. Techniques such as identifying barriers; breaking problems down (into easier chunks) and exchanging information in an ask-tell-discuss manner (e.g. to address misconceptions or to stimulate ideas for overcoming barriers) should have been utilised.

Table 6: Problem solving checklist

Score	Proficiency	Example
	0 – Absence of skill	Absence of discussion to suggest appropriate problem-solving strategies relating to the action plan.
	1 – Novice	Minimal discussion to suggest appropriate problem-solving strategies relating to the action plans and/or inappropriate delivery. Amendments to action plans are not made or made poorly despite being required.
	2 – Advanced beginner	Only a small part of the discussion is delivered to a competent level. Some discussion to suggest appropriate problem-solving strategies relating to the action plans, however, these may not be carried out to sufficient depth or detail. Adapting appropriately to context involved, but numerous problems and inconsistencies present. Detached involvement. Amendments to action plans are made poorly.
	3 – Competent	Competent and numerous discussions to suggest appropriate problem-solving strategies relating to prior action plan, however some difficulties are evident (e.g. opportunities to discuss missed, not all areas of problem covered). Able to cope with different context and situations. Minor problems or inconsistencies present. More engaged involvement. Action plans are amended satisfactorily where required, but problems evident, e.g. goals are no longer SMART.

	4 – Proficient	Numerous discussions to suggest appropriate problem-solving strategies with respect to the action plan, able to discriminate between a variety of contexts and situations with some minor problems or inconsistencies evident. Action plans are amended effectively where required.
	5 – Expert	Highly appropriate suggestion(s) of appropriate problem-solving strategies with respect to problems with the prior action plan. Able to make more subtle refined discriminations between contexts and able to adjust accordingly. Minimal or no discernible problems. Action plans amended without any problems.
	N/A	Not relevant to this activity.

Table 7: Goal amendments

Old Goal or EnviroMod number	New Goal or EnviroMod	Reason for Amendment

Item 2b: Monitoring progress

Intervention techniques

The facilitator should have asked about progress against the action plans made, actively explored areas in which the patient had experienced benefits as a result of their behaviour change and sought to reinforce these benefits in order to help maintain patient motivation and achievement. The facilitator should also have encouraged ongoing self-monitoring of the targeted behaviours.

Table 8: Monitoring progress checklist

Score	Proficiency	Example
	0 – Absence of skill	Absence of discussion to monitor participant progress on the action plan.
	1 – Novice	Minimal or inappropriately delivered discussion to monitor participant achievement on the action plan.

	2 – Advanced beginner	Some evidence of competence. Some discussion to monitor participant progress towards achievement of the action plan, however, not in sufficient detail or depth. Detached involvement.
	3 – Competent	Competent and numerous discussions to monitor participant progress towards the action plan, however difficulties are evident (e.g. opportunities to discuss missed, not covering all aspects of the problem). Competent, aware of and able to cope with different contexts and situations. More engaged involvement.
	4 – Proficient	Involved discussion to monitor participant on the action plan, some minor problems or inconsistencies evident.
	5 – Expert	Highly appropriate and sufficient discussions monitoring participant progress towards the action plan. Minimal problems.
	N/A	Not relevant to this activity.

Item 2c: Managing setbacks

Intervention techniques

Setbacks should have been managed using reframing techniques, to change perspectives to allow for viewing of failures as opportunities for change. Participants should have been informed about coping plans as a strategy for managing the setbacks, to aid in the sustainability of change.

Table 9: Managing setbacks checklist

Score	Proficiency	Example
	0 – Absence of skill	Absence of discussion to review participant setbacks relating to action plans and/or highly inappropriate performance.
	1 – Novice	Minimal (or poorly delivered) discussion to review action plans and/or inappropriate performance.
	2 – Advanced beginner	Some evidence of competence. Some discussion to review participant setbacks relating to physical activity behaviours however, these may not be carried out to sufficient depth or detail. Adapting appropriately to context involved, but numerous problems and inconsistencies. Detached involvement.
	3 – Competent	Competent and numerous discussions to review participant setbacks in achievement of action plans. However, some difficulties are evident, such as minor inconsistencies. More engaged involvement.
	4 – Proficient	Numerous discussions to review participant setbacks when achieving action plans, able to discriminate between a variety of contexts and situations with some minor problems or inconsistencies evident. Some discussion of coping plans.

	5 – Expert	Highly appropriate and sufficient review of participant setbacks relating to achievement of action plans. Able to make more subtle refined discriminations between contexts and able to adjust accordingly. Minor or no discernible problems. Clear discussion of coping plans.
	N/A	Not relevant to this activity.

Item 3: Supporting the Basic Psychological Needs

Key features

The INTEREST study was designed using the theoretical framework of Self-Determination Theory (SDT). Within SDT is the sub-theory of basic psychological needs, which states that we all have three key needs: autonomy, competence, and relatedness. Individuals will maximally achieve behaviour change when these basic needs are most fulfilled. Thus, within the study, all activities should be supportive of the patient's basic psychological needs.

Intervention techniques

To enhance the basic psychological needs throughout the intervention, opportunities should have been taken to enhance participant autonomy by emphasising patient choice (i.e. by emphasising that each of the goals are their choice), highlighting their strengths and agency, and supporting them to overcome their own barriers and to achieve their goals. Competence should have been supported as well, by providing them with the tools they need to feel a sense of achievement, by recognising efforts towards achieving goals, by supporting change talk, and praising participant choices and achievements. Likewise, relatedness should have been aided by fostering an environment in which social interaction can occur, by encouraging spousal/familial involvement where possible, and by having supported or suggested activities that occur in a social context. This included addressing any negative social influences on achievement of the action plans.

Table 10: Supporting Basic Psychological Needs checklist

Score	Quality level	Description of characteristics
	0 – Absence of skill	None of the basic psychological needs were supported.
	1 – Novice	Slight support for one of the basic needs.
	2 – Advanced beginner	The patient was supported in most of the basic needs to some degree, however many opportunities were missed for reinforcement, encouragement, etc.
	3 – Competent	All of the basic psychological needs were supported, but some opportunities to support the patient were missed.
	4 – Proficient	Collaborative relationship between deliverer and patient was clear, and autonomy was highly supported. Few opportunities

		missed to emphasise autonomy, enhance relatedness, or to foster competence.
	5 – Expert	All needs were supported, no opportunities missed.

Item 4: Formulation of an appropriate action plan (action planning)

As the action plans are not purely reliant upon the skill of the deliverer, but also reliant upon the participant having a good understanding of their own context and behavioural patterns, the goal-plan was assessed in two stages. Firstly, quality of support for the action-planning process was self-rated by the facilitator using a measure based on the six-point scale of adult skill acquisition (Dreyfus, 2004) (Table 11). Secondly, plan content and quality was assessed retrospectively at the end of the study. This was conducted with a custom-designed measure of plan content and quality according to the requirements of INTEREST (Table 12), and not the six-point scale of adult skill acquisition. This helped to assess treatment receipt, and goal adherence (self-recorded by participants) helped assess treatment enactment.

Key features

The facilitator should have worked with the participant to formulate an effective set of 6 goals and 3 environmental modifications with which the patient could have reduced their sedentary behaviour and increased their movement. All the goals and environmental modifications should clearly have targeted an aspect of sedentary behaviour or a behaviour with which sedentariness is commonly displaced: namely, they should be related to one of the following:

- Reduction in total sitting time.
- Reduction in the average length of sedentary bouts/greater frequency of breaks in sitting.
- Increased standing behaviour.
- Increased walking.
- Increased quantity of sit-to-stand transitions.
- To increase some other kind of physical activity.

The action plan should also have been appropriate to the level of physical function of the participant. The action plan should have been cross-referenced to the physical function or Short Physical Performance Battery (SPPB) score to ensure that the plan is suitably individualised. Each of the goals should have adhered to SMART principles (i.e. been specific, measurable, achievable, realistic, and timely).

To rate the action plan, the assessor should first have rated each of the goals made for a single participant according to the SMART principles using Table 12 below. Using Table 13 while looking at

the whole of the action plan, plus the scoring in Table 12, the assessor should have then been able to rate the overall level of quality of the action plan. Action plans were considered as delivered to a good level if the average score across the intervention was a 4 or above. Some SMART items, such as whether goals were achievable and realistic may have had to have been assumed or checked against the physical function level of the participant.

Intervention techniques

The facilitator should have ensured that the action plan was made as a collaborative process that was participant-focused and was supportive of their autonomy. The patient should have been heavily involved in the formulation of their own goals, and the purpose of the facilitator was mainly to ensure that the goals formulated are all SMART (as outlined above).

Table 11: Action plan delivery self-rating checklist

Score	Quality level	Description of characteristics
	0 – Absence of skill	No skills were used in the formulation of the action plan.
	1 – Novice	The deliverer dictated most of the goals to the patient with little regard for the patient’s autonomy, and few of the goals meet SMART criteria.
	2 – Advanced beginner	The patient was partially supported in the goal making process. Some of the goals meet SMART criteria. Little evidence of a collaborative relationship between deliverer and patient.
	3 – Competent	The patient and deliverer had evidence of a collaborative relationship. Autonomy of the patient was mostly supportive and feedback was given for the majority of the goals as to whether they adhered to SMART criteria.
	4 – Proficient	Collaborative relationship between deliverer and patient was clear, and the patient confirmed that every goal adheres to SMART criteria. Autonomy of the patient was supported throughout the session. Small issues in delivery still present (e.g. couple opportunities missed to discuss an element of the SMART criteria).
	5 – Expert	Collaborative relationship between deliverer and patient was clear, and the patient confirmed that every goal adheres to SMART criteria. Autonomy of the patient was supported throughout the session. No discernible issues with delivery.

Table 12: Action plan scoring table

Score	Quality level	Description of characteristics
	0 – Non-existent	There is no evidence for an action plan for this participant.
	1 – Inadequate	In total, fewer than 6 goals and/or environmental modifications are created. This could be 3 goals and 2 environmental modifications, for example.

	2 – Adequate	Only one or two items are missing in total. For example, only 5 goals and 2 environmental modifications may be present. Total score for the SMART criteria is above 8.
	3 – Good	All goals and environmental modifications are present and the total score is above 12 on the SMART criteria.
	4 – Very good	All goals and environmental modifications are present, and the goals have been rated and all of them adhere to at least 3 of the SMART criteria, plus a total score of 18 or above for total action plan.
	5 - Excellent	All goals are present, score of 25 or above for SMART criteria, and all are suitable for the physical function level of the participant.

Table 13: Participant goal rating checklist

Goal number	SMART criteria					
	SPECIFIC	MEASURABLE	ACHIEVABLE	RELEVANT	TIMELY	Total score for goal (out of 5)
1						
2						
3						
4						
5						
6						
Total for action plan						
