# Supplementary material for: A novel behavioural INTErvention to REduce Sitting Time in older adults undergoing orthopaedic surgery (INTEREST): results of a randomised-controlled feasibility study
Source: Aging Clin Exp Res. 2020 Jan 23;32(12):2565–85. doi: 10.1007/s40520-020-01475-6 (PMC7680324; doi:10.1007/s40520-020-01475-6)
Supplement: Supplementary file 2 — Supplementary file2 (PDF 898 kb) [file 40520_2020_1475_MOESM2_ESM.pdf]

Qualitative analysis of open-ended adherence comments

A total of 16 participants providing adherence data in the sedentary behaviour booklet also provided qualitative data in the "comments" sections next to the quantitative adherence rating. These varied in length from short phrases or sentences, to entire paragraphs of text. A sample page is shown in the protocol publication (Aunger *et al.*, 2019). The comments provided reflections and reasoning for the quantitative assessments underlying the participants' goal and environmental modification adherence, as well as an understanding of the practical issues that faced participants in the intervention group.

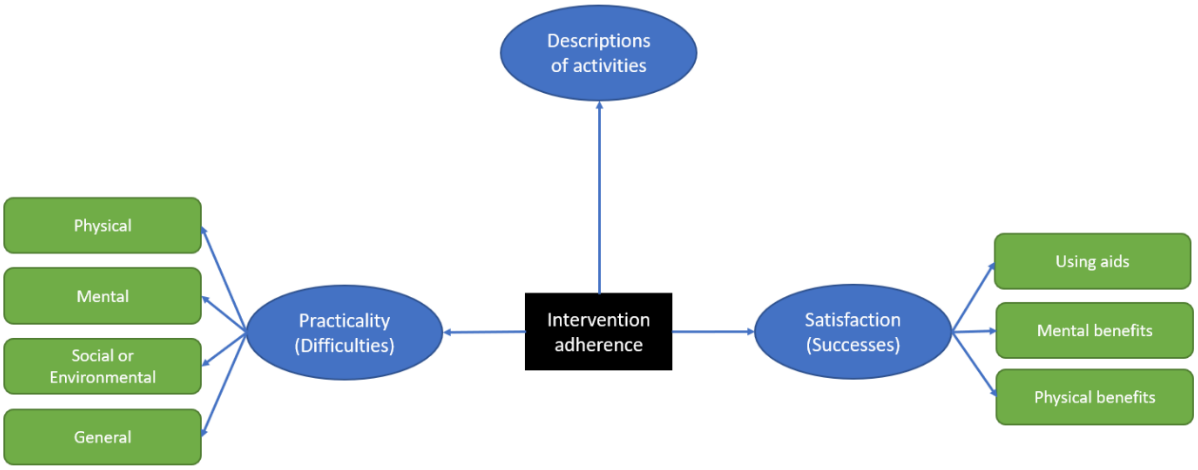

Figure 1. Schema depicting themes and subthemes present in qualitative analysis of participant comments regarding intervention adherence.

Analysis

After a first pass of the transcripts, three main themes were identified in the comments: difficulties, successes, and descriptions of activities. The theme of difficulties included common occurrences that constituted a setback or barrier to achievement of goals; successes included comments regarding perceived benefits to physical or mental health, improvements to motivation, and aids that participants used in achievement of goals they had set; and descriptions of activities included strategies, ideas, and reported behaviours in relation to achieving goals. Figure 2 depicts the number of codes for each difficulty identified by participants.

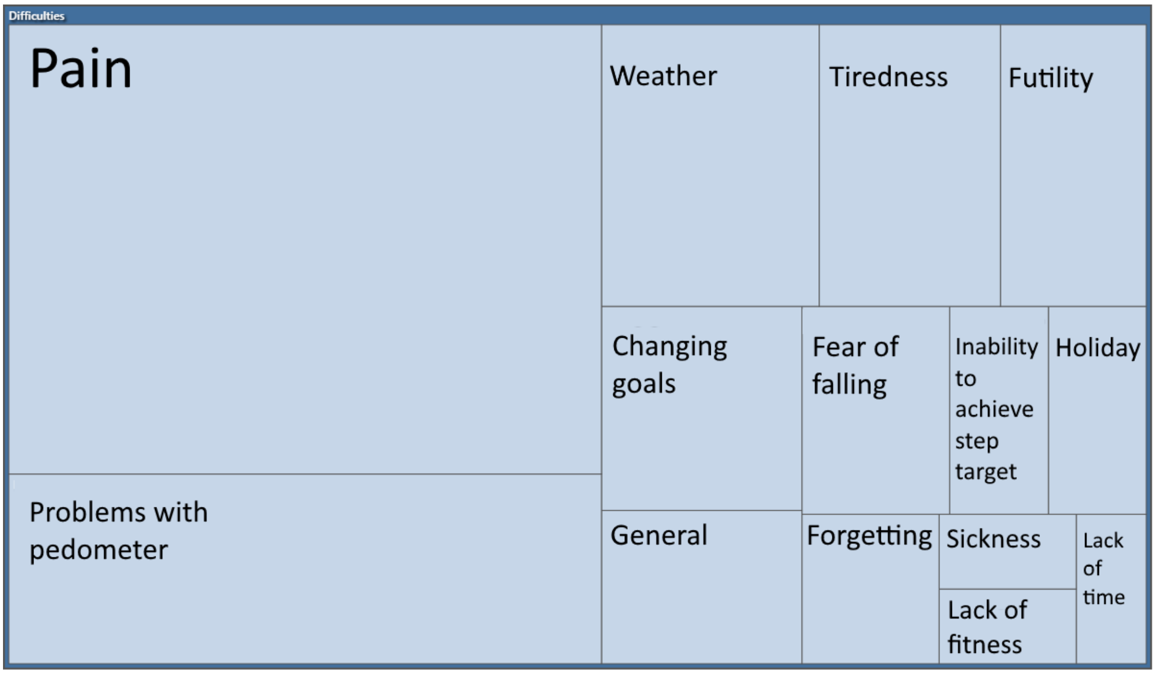

Figure 21. Hierarchy chart displaying the most common difficulties put forward by participants. Size of the boxes represents number of codes.

Practicality (difficulties)

Present in this category were physical, mental, social and environmental, and general subthemes.

Physical difficulties. The largest subtheme within difficulties was pain, comprising 28.3% of total codes. Participants frequently cited pain as the primary barrier to achieving their goals, with 10 out of 16 reporting pain-related problems. Often, participants related it directly to trying to achieve their goals:

> *"I have done this goal for 3 days but found the extra walk led to extra pain" (woman, 68 y, hip replacement)*

> *"Friday night after Rome unable to sleep due to pain – both knees and hip and lower back" (woman, 79 y, knee replacement – after walking on holiday)*

> *"Went shopping on Thursday. I did 1379 steps, but on Friday my hip and knees were aching more" (woman, 71 y, hip replacement)*

Four participants mentioned strategies that could allow them to achieve their goals whilst avoiding some of the resulting additional pain:

> *"Walking not so far, but more often, seemed to be better" (man, 67 y, hip replacement)*

“Struggling with discomfort on walking, finding back pain is increasing, but increased painkiller and plodding onwards” (woman, 67 y, knee replacement)

Additionally, three participants reported that tiredness and lack of physical fitness were something they needed to overcome.

“Don't see how I can reach 3000 steps. Met many goals but not gardening, felt very tired if I did all 6 goals” (woman, 81 y, hip replacement)

“Breathlessness an issue when using slight inclines” (woman, 67 y, knee replacement)

Environmental and social difficulties. The next most common barrier preventing goal achievement was weather. Five participants cited hot, cold, and rainy weather as preventing them from achieving their walking:

“Weather too hot for walking” (man, 79 y, hip replacement)

“Very hard to go into garden in wet. Think of alternative” (woman, 81 y, hip replacement)

“When weather is bad this [goal] is not done, but OK when dry” (woman, 74 y, knee replacement)

Other subthemes were identified on the same level as physical, and environmental and social difficulties subthemes, namely: going on holiday, forgetfulness, a sense of futility or pointlessness to certain goals, fear of falling, and lack of time.

General issues. Another common area of difficulty included problems with the pedometer, the model of which, unfortunately, did not seem to be well-suited to individuals with severe mobility limitations who are not able to walk at pace. Six participants reported issues with the pedometer on this form:

“I now think my shuffling steps do not register - so I am not sitting but not recording movement either” (woman, 81 y, hip replacement)

“Been counting my steps walking around but step counter does not pick up every step” (woman, 71 y, hip replacement)

However, some more technologically minded participants were able to use other solutions to more effectively self-monitor their steps:

"Pedometer didn't operate effectively, spoke to [the researcher] and downloaded [a] health app on [my] mobile" (woman, 67 y, knee replacement)

Satisfaction (successes)

Physical benefits. Although participants predominantly focused on what stopped them from reaching their goals, they also commented on benefits attained from engaging with the intervention:

"Weight loss is continuing very well which does have an impact". "Overall I feel my physical activity is well improved and I feel better for it, have control over pain" (woman, 67 y, knee replacement)

Mental benefits. Mental benefits were also reported, specifically with motivation and additional self-control:

"Feel I have improved and mentally more aware of the benefits after exercise." "Not always 100% happy with results but use it as a motivation" (woman, 67 y, knee replacement)

"Difficult to start, but now motivated" (man, 69 y, knee replacement)

"In pain a lot of the week. Have had to cut walks due to pain but feel better for the movement" (man, 67 y, hip replacement)

Adherence Conclusions

Overall, activity goal and environmental modification adherence means were above 3.9 (out of 5) for both metrics, indicative of good adherence and this was borne out by close mapping of objectively-recorded measures of adherence to step targets. Participants reported their adherence was affected mainly by pain, the weather, and problems with the provided pedometers. To avoid pain while still achieving their goals, some participants used pro-active coping strategies such as increasing painkillers and using sticks. Management of pain and additional fatigue seem to be the main barriers to reducing sedentary time in this population of older adults with osteoarthritis; these are factors that would likely not affect interventions in otherwise healthy older adults. However, when goals were achieved, this conferred a potentially motivational perception of physical and mental benefits to the participants.
