# Supplementary material for: A novel behavioural INTErvention to REduce Sitting Time in older adults undergoing orthopaedic surgery (INTEREST): results of a randomised-controlled feasibility study
Source: Aging Clin Exp Res. 2020 Jan 23;32(12):2565–85. doi: 10.1007/s40520-020-01475-6 (PMC7680324; doi:10.1007/s40520-020-01475-6)
Supplement: Supplementary file 3 — Supplementary file3 (PDF 833 kb) [file 40520_2020_1475_MOESM3_ESM.pdf]

Feasibility - questionnaire (quantitative)

Pre-surgery (Visit 4)

These data are based on responses to the feasibility questionnaire delivered to participants pre-surgery (post-intervention) at Visit 4. These responses comprise were available for 21 people in the intervention group and nine in the usual care group. Completion rate of these questionnaires was 100% for those attending the visit. Means are reported for questions with 1-5 response scales, and medians reported for questions with 1-3 response scales.

Practicality

These questions were all delivered to the intervention group only and assessed practicality.

1. Did you have any problems achieving the goals you set at the beginning of the study? *(scale 1-3: "No problems", "Some problems", or "Many problems").*
   a. Most participants answered that they did have at least have "some problems" achieving the goals they set, giving a median score of 2.

2. Did you have any problems achieving the environmental modifications? *(scale 1-3: "No problems", "Some problems", or "Many problems").*
   a. Most (52.4%) participants reported having no problems achieving their environmental modifications with a median score of 1. This suggests the modifications were easier to achieve than the goals.

3. How difficult was it to achieve your goals physically? *(scale 1-5, 5 is "very difficult")*
   a. This question was asked on a scale of 1-5, where 5 was "very difficult", 3 was "neither easy nor difficult", and 1 was "very easy". The mean answer was 3.29, indicating that participants tended towards answering that goals were difficult to achieve and 42.9% reported achievement being at least "difficult".

4. How easy was it to achieve your goals mentally? *("scale 1-5, 5 is "very difficult")*
   a. This question was asked on a scale of 1-5, where 5 was "very difficult", 3 was "neither easy nor difficult", and 1 was "very easy". Participants tended to report that their goals were mentally "quite easy" to achieve, with a mean score of 2.44. Only 14.3% reported it being mentally difficult to achieve their goals. This indicates goals were physically more difficult to achieve than mentally.

5. Did you find the goals to be well-suited to your individual circumstances? *(scale 1-5, 5 is "very well-suited").*
   a. This question was on a 1-5 scale, with 5 being "Very well suited", 3 "Neither well-suited nor not well-suited", and 1 being "Very not well-suited". The mean answer was 3.95, which indicates that participants found their action plans to be well-suited in general. Only 9.5% reported their goals being "quite unsuitable".

6. How useful did you find the goal booklet? *(scale 1-5, 5 is "very useful")*

a. This question was on a 1-5 scale, whereby 1 was “not useful at all”, 3 was “neither useful nor not useful”, and 5 was “very useful”. 85.7% of participants reported the booklet being at least “useful”, and 42.9% reported it being “very useful”. Only 1 participant found it not useful.

7. Could you have changed your goals to make them more achievable? *(yes or no)*
a. Most participants could not have changed their goals to make them more achievable, however, 33.3% reported that they could have changed them. This indicates that for the most part, participants found their goals to be well-suited.

8. How likely are you to continue working towards your goals in the future? *(scale 1-5, 5 is “very likely”).*
a. This question was on a 1-5 scale, with 1 being “not likely at all”, 3 “neither likely nor not likely”, and 5 “very likely”. Most participants reported continuing to want to work towards their goals, demonstrated by the mean score of 4.5.

Table 1. Questions and responses for questionnaire items that assessed practicality.

**Question**	**Response N (%)**		
	No problems	***Some problems***	***Many problems***
**1**	4 (19.1)	14 (66.67)	1 (4.76)
**2**	12 (57.1)	8 (38.1)	1 (4.8)
**3**	***Answering 1-3, “Very easy” to “neither easy nor difficult”***	***Answering 4+, “Difficult” or greater***	
	11 (52.4)	10 (47.6)	
**4**	18 (85.7)	3 (14.3)	
**5**	***Answering 1-3, “Very not well suited” to “neither well-suited nor not well-suited”***	***Answering 4+, “Quite well suited” or greater***	
	3 (14.3)	18 (85.7)	
**6**	***Answering 1-3, “Not useful at all” to “neither useful nor not useful”***	***Answering 4+, “Quite useful” or greater***	
	3 (14.3)	18 (85.7)	
**7**	***Yes***	***No***	
	14 (66.7)	7 (33.3)	
**8**	***Answering 1-3, “Not likely at all” to “neither likely nor not likely”***	***Answering 4+, “Quite likely” or greater***	
	2 (9.5)	19 (90.48)	

Acceptability

The following questions assessed acceptability and were delivered to both intervention and usual care groups.

1. Have you found taking part in the study burdensome? *(scale 1-5; 5 is most burdensome; both groups)*
a. This question was on a 1-5 scale, with 1 being “not burdensome at all”, 3 “neither burdensome nor not burdensome”, and 5 “very burdensome. Neither group found the study burdensome, with the median answer in both groups being 1. However, 19.0% of participants in the intervention group responded with a score ≥3 (mean: 1.39), versus 11.1% in the Usual Care group

(mean 1.22). The intervention group also had to commit much more time and effort in changing their daily routines. One participant in the Usual Care group rated the study a "3", as the questions in the study exacerbated their ongoing mental health problems.

2. Do you feel that taking part in the study has exposed you to more pain? *(scale 1-3, "no pain", "some more pain", "a lot more pain"; both groups)*
a. The intervention group reported greater increases in pain in comparison to the Usual Care group. The median score in the Usual Care group was 1, and in the intervention group it was 2. This discrepancy is likely due to the intervention group pushing themselves to engage in more physical activity.

3. How likely would you be to suggest taking part in such a study to friends or family? *(scale 1-5, both groups)*
a. One participant reported a "1" as they did not like to recommend participation in such activities in general. The mean in the Usual Care group was 4.88, and 4.28 in the intervention group.

Table 2. Questions and responses for questionnaire items that assessed acceptability.

**Question**	**Response N (%)**		
	Answering 1-3, "Not burdensome at all" to "neither burdensome nor not burdensome"	***Answering 4+, "Quite burdensome" or greater***	
**1 – Intervention**	20 (95.2)	1 (4.8)	
**1 – Usual Care**	1 (11.1)	8 (88.8)	
	No pain	***Some more pain***	***A lot more pain***
**2 – Intervention**	8	12	1
**2 – Usual Care**	9	0	0
	Answering 1-3, "Not likely at all" to "neither likely nor not likely"	***Answering 4+, "Quite likely" or greater***	
**3 – Intervention**	1 (4.8)	20 (95.2)	
**3 – Usual Care**	0 (0)	9 (100)	

Satisfaction

The following questions assessed satisfaction. They were delivered in both groups.

1. How do you feel about being randomised into the group you are in in the study? *(scale 1-5, 5 is "very satisfied", both groups).*
a. Both groups were satisfied or very satisfied with being randomised into their respective groups, with means of 4.4 and 4.8 in the intervention and usual care groups respectively.

2. How would you rate your overall satisfaction with the study? *(scale 1-5, 5 is "very satisfied"; both groups)*
a. All participants in both study arms reported a score ≥4 for study satisfaction, with means of 4.5 and 4.9 in the intervention and usual care groups respectively.

3. *Table 3. Questions and responses for questionnaire items that assessed satisfaction.*

Questions	Response N (%)	
	Answering 1-3, "Not satisfied" to "neither satisfied nor dissatisfied"	***Answering 4+, "Quite satisfied" or greater***
**1 – Intervention**	1 (4.8)	20 (95.2)
**1 – Usual Care**	0 (0)	9 (100)
**2 – Intervention**	0 (0)	21 (100)
**2 – Usual Care**	0 (0)	9 (100)

Safety

The following questions assessed safety in both groups.

1. Do you feel that taking part in the study has exposed you to risk of physical harm? *(scale 1-3; both groups).*

a. Participants did not report that the study exposed them to any risk of physical harm. All participants responded, "no risk".

Table 4. Questions and responses for questionnaire items that assessed safety.

Questions	Response N (%)	
	Answering "no risk"	Answering "some risk" or greater
**1 – Intervention**	21 (100)	0 (0)
**1 – Usual Care**	9 (100)	0 (0)

Post-Surgery (visit 5)

These data are based on responses to the feasibility questionnaire delivered to participants 6-weeks post-surgery, during visit 5. There were 12 responses in the intervention group and 5 in the Usual Care group. Completion rate of these questionnaires was 100%.

Practicality

The following questions assessed practicality and were given to the intervention group only.

1. Since you had your surgery, have you been working towards achieving the goals set in the study? *(scale 1-3).*

a. All participants reported working towards their goals at least "a little".

2. Have you continued to achieve your environmental modifications since surgery? *(scale 1-3).*

a. All participants reported achieving their modifications at least "a little" after surgery. The data may suggest that, after surgery, participants kept working towards goals more so than the environmental modifications.

3. Have you continued to use the sedentary behaviour booklet since surgery? *(scale 1-3).*

a. Most participants seemed to use the booklet not at all, or a little, after surgery.

4. How difficult has it been to work towards the goals set earlier in the study in the absence of the phone calls, etc.? *(scale 1-5, 5 is "very difficult").*

a. Most often, participants reported it being “quite easy” to achieve their goals since surgery. No participant reported it being more difficult than “neither difficult nor easy”. The mean was 2.08.

5. Could your goals have been easier to achieve since surgery? *(yes or no)*
a. Only one participant reported that their goals could have been altered to have been easier to achieve after surgery.

6. How likely are you to continue working towards your goals in the future? *(scale 1-5)*
a. All participants reported being at least “likely” to continue to work towards their goals in the future. The mean was 4.7.

Table 5. Questions and responses for questionnaire items that assessed practicality post-surgery.

**Question**	**Response N (%)**		
	Not at all	***A little***	***Very much so***
**1**	0 (0)	5 (41.7)	7 (58.3)
**2**	0 (0)	7 (58.3)	4 (41.6)
**3**	3 (25)	8 (66.7)	1 (8.3)
	Answering 1-3, “Not difficult at all” to “neither easy nor difficult”	***Answering 4+, “Quite difficult” or greater***	
**4**	12 (100)	0 (0)	
	No	***Yes***	
**5**	11 (91.7)	1 (8.3)	
	Answering 1-3, “Not likely at all” to “neither likely nor not likely”	***Answering 4+, “Quite likely” or greater***	
**6**	0 (0)	12 (100)	

Acceptability

The following questions assessed acceptability and were in both groups.

1. Have you found taking part in the study burdensome? *(scale 1-5, both groups)*
a. Neither group found the study burdensome at this timepoint, however, the intervention group found the study less burdensome at this time compared to the prior timepoint due to the absence of the frequent phone calls and lifestyle changes in this post-surgery period. The means were 1.08 in the intervention group and 1.00 in the usual care group.

Table 6. Questions and responses for questionnaire items that assessed acceptability post-surgery.

**Question**	**Response**	
	Answering 1-3, “Not burdensome at all” to “neither burdensome nor not burdensome”	***Answering 4+, “Quite burdensome” or greater***
**1 – Intervention**	12 (100)	0 (0)
**1 – Usual Care**	5 (100)	0 (0)

Satisfaction

The following questions assessed satisfaction and were in both groups.

1. Do you feel that taking part in the study has influenced your recovery after surgery? *(scale 1-5, 5 is “very positive impact”, both groups)*

a. All participants reported that the study had at least a "positive impact" (a 4 or above) on their recovery after surgery. The mean was 4.5, and the median 4.5 for the intervention group, and 4.25 and 4 respectively for the Usual Care group.

2. How would you rate your overall satisfaction with the study? *(scale 1-5, both groups)*

a. Satisfaction with the study at the post-surgery timepoint was very high, with all participants rating the study a 4 or 5 out of 5.

3. How likely would you be to suggest taking part in such a study to friends or family? (*scale 1-5, 5 is "very likely"; both groups)*

a. Participants were very likely to recommend taking part in such a study to their friends and family. The mean for the intervention group was 4.58 and for usual care was 4.5.

Table 7. Questions and responses for questionnaire items that assessed satisfaction post-surgery.

**Question**	**Response**	
	Answering 1-3, "Very negative impact" to "no impact"	***Answering 4+, "quite positive impact" or greater***
**1 – Intervention**	0 (0)	12 (100)
**1 – Usual Care**	0 (0)	5 (100)
	Satisfaction (mean)	***Satisfaction (median)***
**2 – Intervention**	4.75	5
**2 – Usual Care**	5.00	5
	Answering 1-3, "Not likely at all" to "neither likely nor not likely"	***Answering 4+, "Quite likely" or greater***
**3 – Intervention**	0 (0)	12 (100)
**3 – Usual Care**	0 (0)	5 (100)

Safety

The following question assessed safety and was only in the usual care group.

1. Do you feel that taking part in the study has exposed you to risk of physical harm? *(scale 1-3, "no risk", "some risk", "a lot more risk").*

a. All participants answered 1, "No risk", for this question.

Table 8. Questions and responses for questionnaire items that assessed safety post-surgery.

**Question**	**Response N (%)**		
	No risk	***Some risk***	***A lot of risk***
**1**	5 (100)	0 (0)	0 (0)

Quantitative feasibility results from feasibility questionnaires

Questions were analysed according to the timepoint they were delivered at (either visit 4 or 5) and group. 30 participants answered at visit 4, and 17 at visit 5. In visit 4 (pre-surgery/post-intervention), participants in the intervention group reported having some problems achieving their goals in general, but no problems with the environmental modifications. They also found goals difficult to achieve physically, scoring a mean of 3.29 out of 5, but did not find it as mentally taxing. Participants reported their goals being well-suited on average, and reported wanting to continue to work

towards them in the future. For questions delivered to both groups, the intervention group reported it being slightly more burdensome, but neither found it very burdensome overall. The intervention group also reported enduring more pain due to the study, which is likely due to them attempting to increase their activity. Both groups were very satisfied with the study, reporting means of 4.4 in the intervention and 4.8 in the usual care groups (out of 5). Neither group reported feeling at risk of physical harm.

At post-surgery (visit 5), all intervention participants reported continuing to work on achieving goals set in the study at least “a little”, but not so much for the environmental modifications. The study was less burdensome at this timepoint to both groups, and both groups thought the study had a positive impact on their recovery (means of 4.5 in intervention, and 4.25 in usual care group). All participants rated satisfaction of the study as a 4 or 5 out of 5.
