# Supplementary material for: A novel behavioural INTErvention to REduce Sitting Time in older adults undergoing orthopaedic surgery (INTEREST): results of a randomised-controlled feasibility study
Source: Aging Clin Exp Res. 2020 Jan 23;32(12):2565–85. doi: 10.1007/s40520-020-01475-6 (PMC7680324; doi:10.1007/s40520-020-01475-6)
Supplement: Supplementary file 4 — Supplementary file4 (PDF 802 kb) [file 40520_2020_1475_MOESM4_ESM.pdf]

Feasibility - questionnaire (qualitative)

Feasibility data were coded using deductive, realist thematic analysis (Braun, Clarke and Weate, 2016), because themes were pre-specified according to feasibility criteria specified in the protocol and informed by Bowen et al. (2009), namely: acceptability, adaptation, practicality, safety, and satisfaction and feedback. However, subthemes were inductively created based on patterns within the responses by the participants during the familiarisation process. Top-level themes were based on feasibility criteria, however, generation of initial subthemes was based on a first-pass of the data, followed by review and refinement of these codes (Braun and Clarke, 2006). Data are presented per-theme and elements highlighted where there were differences between groups and timepoints.

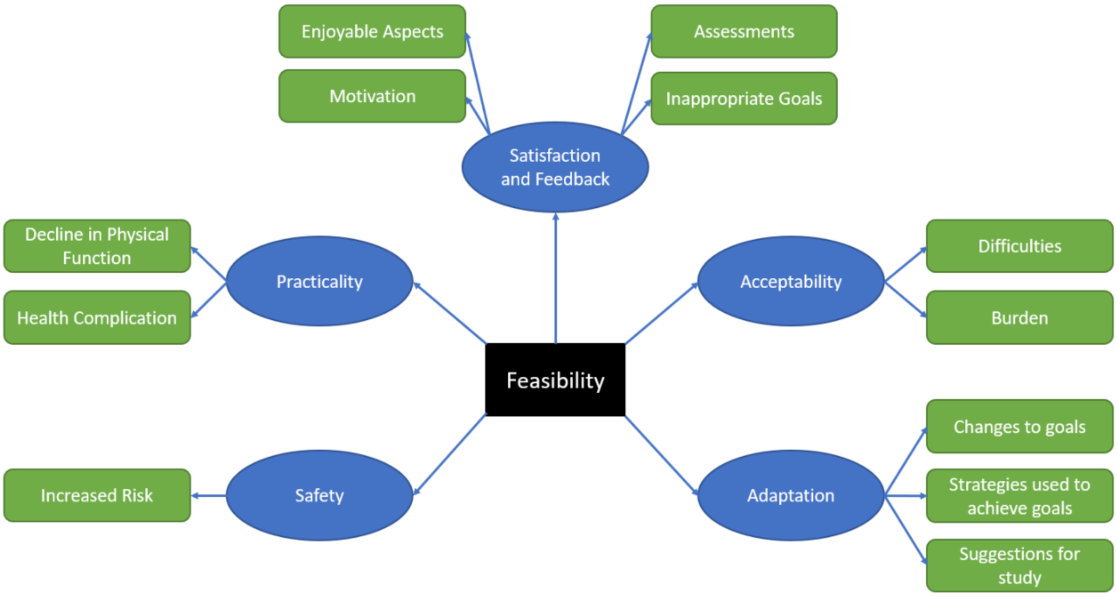

Figure 1. Schema depicting themes and primary sub-themes arising from thematic qualitative analysis of feasibility questionnaire data.

Questionnaire items stratified by feasibility elements

All questions on the questionnaire were intended by design to correspond to different feasibility themes. However, some participants wrote answers to some questions that did not fit into the intended theme for the question (table 1), and thus, were coded to another theme instead. Table 1, following, depicts the overall planned areas covered by each of the questionnaire questions at each timepoint.

Table 1. Questionnaire feasibility themes.

Feasibility Theme	Question
Pre-Surgery	
Acceptability	1. Which part(s) of the intervention did you find most difficult?

Practicality	2.	If you found it burdensome, how could it have been improved? *(both groups)*
Satisfaction	3.	Which part(s) of the intervention did you find most enjoyable?
Adaptation	4.	Do you have any suggested improvements for the booklet?
	5.	Is there anything you would change about the assessments? *(both groups)*
	6.	Do you have any suggested improvements for the study?
Safety	7.	If you found an aspect of the study more painful or harmful than usual, how? (*both groups*)
Post-Surgery		
Satisfaction/Practicality	1.	How have you found the assessments in the study so far (e.g. the questionnaires, physical tests, etc.)? *(both groups)*
Adaptation	2.	Do you have any suggested improvements for the study? *(both groups)*
	3.	Is there anything you would change about these assessments? (*both groups*)
Safety	4.	If you found an aspect of the study more painful or harmful than usual, how? (*both groups*)

Analysis

Acceptability

Burden. As seen in the quantitative data regarding burden, overall burden was higher in the intervention group. Only one participant in the Usual Care group commented at the pre-surgery timepoint that *"due to mental issues"*, partaking in the study was *"too much" (*man*, 67, knee replacement).*

Difficulties. Most participants reported some in achieving the intervention goals. Pain and weather, as seen in the adherence analysis, were common barriers, with participants reporting that *"the pain barrier has to be overridden" (*man*, 87 y, knee replacement),* as well as social responsibilities: "*social responsibilities/commitments and some weather condition interruptions" (*woman*, 67 y, knee replacement).* There was a wide range in what was purported to cause difficulty, ranging from *"attaining the steps"*, to *"opening the pedometer"*, as the pedometer had a screen that was difficult to gain access to in individuals with arthritic hands. Others also mentioned that *"the step monitor failed after [2] weeks (so I purchased a monitor)"*, and that *"it did not work with the steps I now take (a slow shuffle). This made keeping the record of steps inaccurate" (*woman*, 81 y, hip replacement).* These issues suggest that the pedometer was not friendly for the older people in the study who walked at a slower pace.

A number of participants (3) also stated that the chair rises were most difficult, as some formulated goals to do several of these in sequence at certain times of the day. Other specific goals were mentioned as causing difficulty, such as *"standing for breakfast, leaving TV until after 9pm" (*woman*, 72 y, hip replacement), "remembering to stand up whilst on the phone" (*woman*, 79 y, knee*

replacement), and *"using the exercise bike" (wo*man*, 68 y, hip replacement).* Certain times of day were also more difficult for some people, with *"evening exercise"* being specified as being difficult, due to fatigue and a desire to relax in the evening.

Others reported mental aspects being the most difficult to contend with, reporting *"more self-discipline and better weather"* would have improved things for them, or simply that *"motivation"* in general was the most difficult aspect of the study for them.

Pain on exercising was a further barrier for many participants:

> *"My right knee became much worse so I had to rest for a few days on advice from my doctor" (woman, 66 y, knee replacement)*

Another found that implementing their goals led to greater pain:

> *"I found taking the dog for an extra walk made my pain worse, as did using the exercise bike" (woman, 68 y, hip replacement)*

One participant mentioned that, in relation to pain, "*evening activity is worst to achieve*" (man, 74 y, knee replacement).

Practicality

Decline in physical function. One participant mentioned that from baseline to pre-surgery, *"getting up from chairs/toilet became progressively more difficult" (wo*man*, 79 y, knee replacement)*. This may be because some participants were increasingly feeling the impact of arthritis over time or many other reasons.

Health complications. Five participants in the intervention group mentioned other health conditions as well as consequences of the operation that had affected achievement of their goals. One mentioned the following after surgery:

> *"I have not achieved all my goals since surgery because I have quite severe oedema. My doctor is investigating - apparently I am anaemic and have low levels of protein in the blood. I also have problems with low blood pressure." (woman, 79 y, knee replacement).*

Others also had additional joints that still had osteoarthritis (other than the one to be operated on during the study) that also needed to be replaced, which affected them both in pre-surgery and post-surgery phases:

> *"I am waiting to have my other hip replaced which is more problematic, so cannot walk without aids" (man, 79 y, hip replacement).*

One participant mentioned that they found the study "*burdensome because of other difficulties I have had with severe shoulder pain due to a tear in my tendon*" *(woman, 73 y, hip replacement).* Another couldn't keep up with the goals of the study due to being "*unwell for two weeks*" *(woman, 74 y, knee replacement)*.

Health issues arose during the study that also affected goal achievement, which should have led to greater action by the researcher to alter their goals. One participant mentioned that *"because of my other health issues I found my goals should have been changed half way through"* and said that it was difficult *"keeping to the goals first set due to my other health issues" (woman, 73 y, hip replacement)*. This indicates that in some cases, goals should have been adapted//refined following review of progress but were not.

Satisfaction and feedback with the study procedures

Assessments. For assessments, participants mentioned that they "*find questions difficult*" and that they had "*lots of repetitiveness*", and that "*questions [were] sometimes ambiguous and could be answered in many ways depending on one's interpretation*" *(woman, 67 y, knee replacement).* Additionally, one mentioned that the physical tests were not so applicable to the daily life of older people, and that "*it would be good to have a wider range of physical tests, e.g. put in some housework and general activity tests*" *(woman, 81 y, hip replacement).* However, overall, there were more positive comments than negative. One individual had a number of comments to make about the assessments:

> *"i) Designed to promote self-awareness. Ii) Responsibility for promotion of self-betterment. Iii) Encouraging. Iv) Hopefully promote academic success" (man, 70 y, hip replacement)*

Some people enjoyed the psychological aspects of the assessments, as they made them self-reflect, and *"found them interesting and achievable" (woman, 68 y, hip replacement)*. However, the following comment was from an individual in the Usual Care group:

> *"Made me think how much time I sit. Been very useful. Felt very relaxed with the study" (woman, 69 y, knee replacement)*

This comment suggests that, even in the Usual Care group, the additional self-reflection caused by the assessments alone could have the potential to go on to affect behaviour.

Dissatisfaction. One participant was unhappy with being in the Usual Care group and *"would have preferred to be more 'active'" (man, 70 y, hip replacement).*

Enjoyable Aspects. There was great variation in what participants found enjoyable about the study. Many participants mentioned mental benefits, such as increasing awareness and self-reflection in terms of being *"more aware of activity levels" (man, 73 y, knee replacement)* or that it gave *"confidence in achievement, helped me concentrate on doing more" (man, 79 y, hip replacement).* According to another, it also enhanced *"natural daily activity and being conscient of movement – more focus and awareness" (woman, 81 y, hip replacement)*. In some, it helped them realise for the first time the importance of physical fitness:

> *"Changing attitude towards the phrase fitness/activity which I began to realise were different. It was good to find myself 'fitter' rather than chasing being 'active'" (woman, 67 y, knee replacement).*

Participants seemed happy with the goals, and how they provided "*targets to walk towards, e.g. getting up from a sitting position without using hands*" *(woman, 79 y, knee replacement)*. Another mentioned that "*aims set were appropriate to stretch me and motivate me to take a step back and look how I had let my physical side deteriorate. These issues I was able to address*" *(woman, 67 y, knee replacement)*.

A few were also happy with social benefits, whether it was to have the chance to talk to the researcher, saying that *"chatting to the researcher"* was also a benefit of taking part in the study, or with family and friends. Another said that benefits were obtained in the form of *"encouragement from partner and family. Study has provided me with an outlook to discuss with others." (man, 79 y, hip replacement).*

Physical benefits were also mentioned, as well as the positive effect of resulting successes on perceptions of strength, mobility, and general wellbeing. One participant mentioned that their *"legs have become stronger"*, and another that they were *"glad to have achieved the chair rises. Glad to be a bit more active" (woman, 71 y, hip replacement)*. One participant mentioned at post-surgery that "*It has been very helpful considering other operations I have had and time it has taken for recovery" (woman, 74 y, knee replacement)*. One participant felt no need to use their mobility aid any longer:

> *"The fact that a short time into the intervention I was able to walk further and felt much more mobile. I have not used my walking stick once since starting the study - great!" (woman, 68 y, hip replacement).*

Using achievement as a motivation was a common theme, as was reported by these participants:

> *"I found it great taking a look at myself and the achievements I have made (I really enjoyed the pedometer)" (woman, 66 y, knee replacement).*

> *"Initially [the main benefit] was achievement. It became obvious that I was not as active as I thought I was and needed to do more 'physical activity' on a daily basis" (woman, 67 y, knee replacement).*

People also found specific activities particularly enjoyable, rather than any benefits attained. Just as some said *"chair rises"* or the pedometers as the main difficulties, others mentioned them as aspects they enjoyed.

Motivation. Motivation was also mentioned as being positively affected. One participant mentioned that it *"gave me motivation to look round at myself and to start to get things in order, make changes" (woman, 79 y, hip score)*, and another that it *"purely motivated me to push harder and get overall fitness higher" (woman, 67 y, knee replacement).*

Adaptation of the study

Changes to goals. Participants made changes to their goals throughout the study, which they mentioned in the questionnaire. Most common was reducing the step count, specifically *"to make them more achievable" (woman, 74 y, knee replacement)*, and, in another case, to *"stop when it hurts" (man, 67 y, hip replacement)*. One other person wanted to start doing *"sit to stands each day"* of their own volition, suggesting good engagement with the notion of increasing their activity.

Adaptive strategies used to achieve goals. It became evident from responses that participants in the intervention group adopted several strategies in the achievement of their goals, both at pre-surgery (Visit 4) and post-surgery (Visit 5) timepoints. At Visit 4, participants mentioned *"when the pain was too severe, I did exercises sitting down, lowering myself to the chair slowly gave less pain" (man, 87 y, knee replacement)*. One participant had a goal of 5 chair rises after every meal, however, they commented that they *"could have done chair rises before meals to avoid indigestion" (woman, 77 y, hip replacement)*. Further participants used different strategies to achieve the purpose of their goals. In one case, they mentioned that *"[keeping the] TV remote [far away] was not done, but used timer instead" (woman, 77 y, hip replacement)*, thus still prompting themselves to stand up whilst watching TV. At Visit 5, a participant mentioned that they *"should have used the pedometer more because it helped me to walk more" (man, 73 y, knee replacement)*, indicating that they regretted not keeping up with it since surgery.

Suggestions for the study. Participants mainly had suggestions for the assessments in the study and the booklet (other than issues with the pedometers, mentioned under "difficulties"). For the booklet,

participants mostly found it to be good, however, a few mentioned the "worksheets" section to be confusing and needed *"more writing room"* to be able to properly fill it out.

5.2.5. Safety

Increased risk. One participant mentioned that one of their goals made them afraid of falling, saying that "[the] target of gardening was not a good choice (too problematic underfoot). Did not feel safe" *(woman, 81 y, hip replacement)*. This suggests this goal could have been changed as it was not realistically achievable.
