# Supplementary material for: A novel behavioural INTErvention to REduce Sitting Time in older adults undergoing orthopaedic surgery (INTEREST): results of a randomised-controlled feasibility study
Source: Aging Clin Exp Res. 2020 Jan 23;32(12):2565–85. doi: 10.1007/s40520-020-01475-6 (PMC7680324; doi:10.1007/s40520-020-01475-6)
Supplement: Supplementary file 5 — Supplementary file5 (PDF 998 kb) [file 40520_2020_1475_MOESM5_ESM.pdf]

Feasibility of recruitment (interview with RNs)

A single interview was conducted with the primary research nurse assigned to the project in March 2019. A second research nurse was not able to perform significant duties on the study due to an unexpected leave of absence from work, so only one interview was performed. The purpose of the interview was to assess satisfaction, practicality and ideas for adaptation of the recruitment process in INTEREST, from the perspective of the primary deliverer of recruitment.

Results

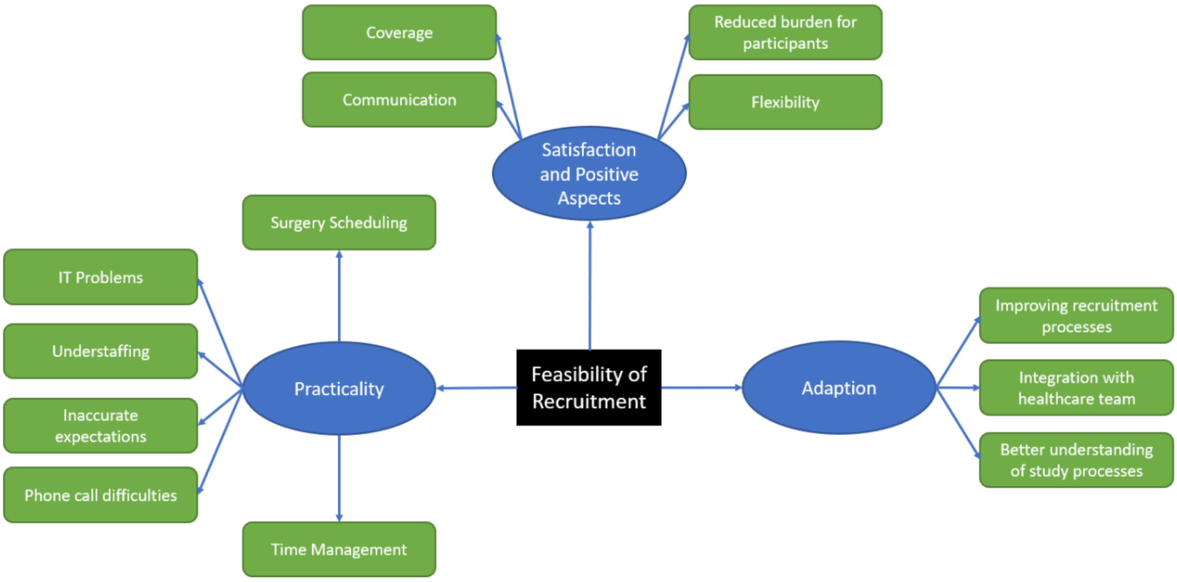

Figure 1. Schema of themes and subthemes relating to the feasibility of recruitment resulting from the qualitative analysis of the interview with the research nurse.

Context

The research nurses in this study performed several tasks, including managing most recruitment-related processes, and dealing with communication from the research team regarding surgery scheduling. The recruitment process involved sending out participant information sheets to participants approximately 10-12 weeks before surgery, which required knowledge of an estimated surgery date. Approximately a week after the PIS was sent, the RN could also call the participant directly to ask if they were interested in taking part in the study.

When sending the PIS, the expectation was that patients would have surgery approximately 16 weeks after seeing the clinician for the first time, so this date was used for surgery date estimation. After this point, the RN could request updated dates for surgery from the clinicians' secretaries, if asked to do so by the researcher.

Interview findings

Headings indicate the themes being discussed, and bold text indicates the subtheme.

What were the problems that arose during recruitment? (Practicality)

Several problems with recruitment were identified. Not all problems presented here were directly related to recruitment, but also to study processes in general. These included: problems related to understaffing, inaccurate expectations, IT problems, problems relating to phone calls, time management, and problems with surgery scheduling.

Problems relating to understaffing. As the second RN assigned to the project had an unforeseen leave of absence, this led to a significant burden on the primary RN and an increase in their workload. The RN mentioned that this led to increased burden, as sending out PIS' *"would take like a whole day"*, and if the other RN was present, then *"we could have done it a bit differently"*. However, they did not report that this impacted recruitment, just made it more difficult, as *"we've definitely picked up everyone that we could have picked up, everyone's had the opportunity"*.

Inaccurate expectations. At the site initiation visit, the researcher was informed that it was very likely that the target of 45 participants over one year would be achieved. This was based on the number of eligible patients likely to be listed for surgery in the period. However, the RN reported that *"there weren't as many responses as I thought to come back"*.

IT problems. The RN initially had problems accessing surgery dates directly, which negatively impacted recruitment up until 04.05.2018, when the issue was resolved. The RN reported that: "*initially I couldn't do it myself on the IT system, so I was having to email the secretaries to send me their updated theatre lists, which obviously wasn't as live as if I could just do it every day, if you know what they mean, and some of them were reluctant in sending it, or just not sending it. So that was a delay at the start, but when I was able to get access it was sorted."*

Problems relating to phone calls. Two issues were reported with the phone calls, firstly, that *"they probably weren't done the week after if you know what I mean, just because of workload and other stuff in the office or whatever…"*. Additionally, the RN reported that there were wrong numbers in the patient records, and that many didn't answer. This meant that the RN *"did manage to speak to quite a few, but yeah, not all of them, really"*.

Problems with time management. The RN reported throughout that it was a time-intensive process to do the recruitment tasks, both the phone calls and sending out PIS'. In relation to the study, they mentioned that when they were busy, *"you could say [the study] was pushed to the side if you know what I mean – but it wasn't delayed"*.

Problems with surgery scheduling. Towards the end of the study, and over the summer 2018 period, there were lulls in recruitment. The RN reported that the problem times were *"Christmas! Christmas! And, weirdly, summer, because of all the holidays that people have – both patients and – so they start to on the waiting list you can see that they can't have their surgery between here and here, so it gets delayed and stuff by the patients"*. Evidently, one of the reasons for these delays were holidays. However, the RN also mentioned that there were problems with the surgical theatres over the Christmas period: "*theatre 10 has got some kind of problem, and as such they've opened up an outdoor thing, some kind of Vanguard unit. Could that have affected [scheduling]? Yes. Whether it did? I don't know."*

How could recruitment have been improved?

The RN identified a few areas for improvement, these included improving recruitment processes, improved integration with the healthcare sector, lengthened recruitment times, and giving RNs a better understanding of study processes.

Improving recruitment processes. The RN suggested that we could improve the uptake rate by approaching participants face-to-face, to *"see a few patients in the hospital and tie it in with the initial clinic day, almost have a stand with posters or something, and just say if this would be something you'd be interested in once they were listed for theatre and we know you've got a date"*. The RN believed that simply sending the PIS was too passive and that it *"would have been better if we were able to see them physically rather than it just being sent out"*. Likewise, the PIS material was too long and needed to be cut down. According to the RN, based on phone calls they had experienced, *"some of [the patients] just didn't want to know just because of the amount they were being sent"*, which could have affected the uptake rate more. A further perspective resulting from the phone calls was that the participants would have preferred to talk to the researcher, as the researcher was better informed regarding the study processes and *"could say [I am] the one who would directly come out to see them"*.

Prolonged recruitment times could mean more patients could have been followed-up due to long surgery delays. The RN suggested that although we *"need a time limit when you're trying to recruit these patients, but if you had two years, then you wouldn't have had [so many issues]"*.

Integration with the healthcare team. When asked about integrating the research team with hospital processes more, the RN thought that *"if you could have better access at whatever hospital you were at, just so if you could even remotely access the system and stuff, yeah, that would be good"*. Direct access to surgery lists by the researcher could streamline research processes but may present ethical issues.

Better understanding of study processes. The RN reported that "it could have been quite good to have seen a visit, maybe, it just might have been useful to see what it fully entailed, as you understand it quite a bit better then, rather than saying 'I think this is going to happen', and you know". As over 99% of study visits were scheduled at home, it wasn't possible to coordinate one with the research nurses, but this would have enabled them to explain the study on the phone to participants more effectively.

How would such a study scale to another research site or a definitive trial?

The RN reported that expanding the study to another research site would likely come across the same issues with respect to surgery scheduling, as they *"assume it would be the same, as they all have the same 18-week wait or something"*. Furthermore, they responded affirmatively to the question of whether other hospitals "would all suffer from the same bed shortages".

What were the positive aspects of the design of the INTEREST feasibility study?

The RN thought that the approach to recruitment, although flawed in some ways, was beneficial in others, as outlined further below.

Coverage. Although not everyone wanted to take part, *"we were capturing the right patients that were supposedly going to be seen"*.

Communication. With respect to communication between the RN and the research team, the RN thought it was good. Although having direct access by researchers to surgery lists would make processes more fluid, the RN thought that *"the communication [between RN and researcher] was the most information you could have had"*.

Flexibility. The RN was also happy about the flexibility that the recruitment processes offered, as they could send out PIS' and make calls around their other commitments.

Burden. The RN was of the opinion that the study did improve uptake rate as *"[the researcher] definitely picked up more people because of going to their houses"*, which reduced participant burden.

Summary

The primary roadblocks affecting practicality that were perceived by the RN were problems with operating theatres and the length of participant-facing documents. Recruitment could be improved by reducing the length of these documents, increasing the recruitment period and overall study length, and by affording the RNs a better understanding of activities within study visits. For adaptation, the RN does not foresee any issues with scaling to other research sites, other than that the same caveats found here are likely to remain. Lastly, for the category of satisfaction, they found that the

recruitment processes in INTEREST were positive since it managed to reach all eligible patients, and the visits in participants' homes likely improved uptake rate due to reducing burden.
