# Supplementary material for: A novel behavioural INTErvention to REduce Sitting Time in older adults undergoing orthopaedic surgery (INTEREST): results of a randomised-controlled feasibility study
Source: Aging Clin Exp Res. 2020 Jan 23;32(12):2565–85. doi: 10.1007/s40520-020-01475-6 (PMC7680324; doi:10.1007/s40520-020-01475-6)
Supplement: Supplementary file 6 — Supplementary file6 (PDF 473 kb) [file 40520_2020_1475_MOESM6_ESM.pdf]

Analysis of missing data and feasibility of data collection

Baseline

Data collected from questionnaires and physical measurements at baseline were complete in both the 24 individuals in the Intervention group, and the 11 in the Usual Care group, indicating a high degree of feasibility for collection of this data type.

Blood sampling

There were issues present with collecting blood samples, which meant that completeness for certain measures ranged from 81.8% (9) in the Usual Care group for red blood cell concentration, to 77.7% (7) for cortisol concentration. This discrepancy in number between measures is primary due to issues with analysis at the University Hospitals Birmingham Clinical Laboratory Services (UHB CLS), whereby results of certain analyses were not returned to the researcher, without reason being given, causing missing data for certain biomarkers. However, the missing 2 cases in the Usual Care group were due to inability to find a vein by the primary researcher. As phlebotomy was conducted in people's homes, it was not possible to have a backup nurse take the sample, so three attempts were made and then attempts were ceased. In the intervention group, there was a similar discrepancy; with 95.8% (23) of values being present for vitamin D, but only 91.6% (22) present for cholesterol, LDL, and others. This was also due to issues with analysis, most often due to results not being returned to the research team or an insufficient sample remaining after the initial analyses.

ActivPal measurements

ActivPal measurements were also not complete, with 90.9% (10) of usable (defined as >2 valid days of recording) data present in the Usual Care group, and 91.7% present in the intervention group. In the Usual Care group, the n=1 missing case was due to data corruption, and in the intervention group, the missing cases were due to 1 data corruption, and 1 drop out/non-return of the device.

Pre-Surgery (Visit 4)

The majority of data was feasible to collect, with little missing data present in the n=21 (Intervention) and n=9 (Usual Care) individuals that reached this timepoint. One pre-surgical meeting in the Usual Care group had to be cut short due to a participants' mental health issues, which led to missing data (n=1) being present for SPPB, WHR, weight, BMI, IPAQ, blood measures, and Oxford joint score. However, other questionnaire-based data were recovered via a follow-up phone call.

Blood sampling

The most missing data, again, occurred with blood sampling. The Usual Care group had 77.8% (7) of data present for cholesterol, LDL, triglycerides, etc., however, only 55.5% (5) of data was present for

cortisol, for example. This discrepancy was again due to analysis issues at UHB CLS. However, it was also not possible to gain blood samples from 2 participants.

In the intervention group, blood sample data was present in some level of completeness for 81.0% (17) of participants. This again varied from 17 cases for red blood cell count, but only 15 for cortisol. The discrepancy within subjects was again due to analytical issues, however, the missing 4 subjects were due to insufficient time to ask the participant to fast (1), difficulty in blood draw (2), and refusal (1).

ActivPal measurements

Only 66.7% (6) participants in the Usual Care group and 95.2% (20) of those in the intervention group provided sufficient ActivPal data for analysis pre-surgery. It was difficult to acquire all data at this timepoint as measurements had to occur during the week preceding surgery (in those who had surgery), and if data were corrupted or lost power too quickly, then measurements could not be re-attempted. Data were missing in the Usual Care group due to non-return of ActivPal (1), data corruption (1), and battery failure (1). In the intervention group, one was missing due to insufficient time to record (1).

Post-Surgery (Visit 5)

All data for physical measures (weight, SPPB, etc.) and questionnaires (EQ-5D-5L, MOST, etc.) were present for the 5 individuals in the Usual Care group and the same applied for the 12 individuals in the intervention group.

ActivPal measurements

Data were present for all 5 individuals in the Usual Care group, but only 66.7% (8) of the intervention group. This is due to incorrect wear (1), data corruption (2), and battery failure (1).

Summary of analysis of missing data and feasibility of data collection

It was feasible to collect all the data for this intervention, however, battery, lack of time for sufficient measurement, and other technology issues affected ActivPal data completeness, and a lack of backup personnel and analytical issues affected blood measure completeness.
