# Supplementary material for: A novel behavioural INTErvention to REduce Sitting Time in older adults undergoing orthopaedic surgery (INTEREST): results of a randomised-controlled feasibility study
Source: Aging Clin Exp Res. 2020 Jan 23;32(12):2565–85. doi: 10.1007/s40520-020-01475-6 (PMC7680324; doi:10.1007/s40520-020-01475-6)
Supplement: Supplementary file 7 — Supplementary file7 (PDF 638 kb) [file 40520_2020_1475_MOESM7_ESM.pdf]

Intervention fidelity

Treatment delivery (motivational interview skill usage)

Twenty-one motivational interviews (MIs) were conducted, as one participant dropped out of the study prior to the motivational interview. One third (33.3%) of these interviews were recorded and rated for fidelity by two raters (self-rated by primary researcher and one independent expert). A further one was self-rated only.

Table 1. Treatment delivery skill assessment.

	MI Skills	Supporting Basic Psychological Needs
**Self-rated (n=8)**	3.63(0.48)	3.38(0.48)
**Independently rated (n=7)**	3.43(0.53)	2.07(0.63)
**Total mean**	3.53	2.73

While MI quality skill ratings were close, ratings of supporting the basic psychological needs were less congruent. There may be a few reasons for this. Firstly, this methodology had several limitations. The primary issue was that the MI recording only covered the initial aspect of the discussion in visit 2, the formal motivational interview. As such, the independent rater is rating only the first part of the participant visit, while the primary researcher self-rated the entire visit, which also consisted of action planning. The action planning phase contained further use of the supporting basic psychological needs skill that was not accounted for in the recording of the MI session, which was likely to have contributed to a discrepancy in ratings for the supporting basic psychological needs skill.

Treatment delivery (phone call skill usage)

A total of 75 phone calls were delivered as part of the intervention (3 calls each for 25 participants). One individual dropped out after randomisation but prior to receiving the first phone call. Of these, 33.3% (n=25) phone calls were self-rated for fidelity by the deliverer. Goals were changed as a result of the phone calls in 12% of cases. Additionally, for some skills it was determined that they did not need to be used (i.e. patient didn't report any setbacks), so the "times not used" row reflects the occurrence of this. Fidelity ratings for skills used in the phone calls can be seen in the following table:

Table 2. Phone skill usage assessment.

	Monitoring Progress	Managing Setbacks	Problem Solving	Motivational Interviewing	Supporting Basic Psychological Needs	Total
**Mean (SD)**	3.88(0.82)	3.38(1.05)	3.81(0.85)	3.75(0.83)	3.92(0.69)	3.79(0.19)
**Median**	4	3	4	4	4	4

Times not used	0 (0%)	4 (16%)	4 (16%)	9 (36%)	0 (0%)	n/a

Overall, the phone calls were delivered to a competent standard or greater, with some to "proficient". However, these ratings may be overestimated due to the impact of self-rating bias. Determinations of when certain skills were not required were made entirely by the deliverer, hence certain skills may have not been used when it would have been more efficacious if they were.

Treatment receipt (ratings of action plan)

Action plans were rated by the main researcher as well as an independent rater according to the complete intervention fidelity assessment guide criteria, available in the supplementary files of the published protocol. This constitutes rating all 132 goals for SMART criteria (Specific, Measurable, Achievable, Relevant, and Timely) and giving a total score out of 30, and then assessing the action plan for completeness and appropriateness to the physical function of the participant on a scale of 1-5.

Table 3. Action plan quality assessment. Data are mean (SD).

Goal Rating Scores		Overall Action Plan scores	
Rater 1	Rater 2	Rater 1	Rater 2
27.91 (1.28)	28.18 (1.77)	5 (0.00)	5 (0.00)

Overall, the action plans were assessed to have been constructed to a high standard. As such, it is unlikely that the results of the intervention would be negatively affected by the action plan formulation. The high ratings are likely due to these plans being actively checked by the researcher during their construction.

Treatment enactment

Treatment enactment was assessed with recording of goal adherence, which was already covered in the feasibility section.

Fidelity Summary

A mean score of all skills used was computed, giving equal weighting to all skills and incorporating the independent rater for MI sessions equally as well. Overall fidelity of treatment delivery in the study was competent, with a mean of 3.12 (out of 5). According to the pre-defined criteria, all aspects of the study must have been delivered to the standard of 3 or above to not call into question standard of the delivery of the study. The mean of the independently-rated and self-rated scores for supporting basic psychological needs during the MI session was 2.73 (out of 5), which may indicate that this aspect was (slightly) sub-optimally delivered, which could negatively impact the results of the study.

Quality of action plans was delivered to a good standard according to both the primary and independent rater, with all plans being rated at 5, the maximum score.
