# Supplementary material for: A novel behavioural INTErvention to REduce Sitting Time in older adults undergoing orthopaedic surgery (INTEREST): results of a randomised-controlled feasibility study
Source: Aging Clin Exp Res. 2020 Jan 23;32(12):2565–85. doi: 10.1007/s40520-020-01475-6 (PMC7680324; doi:10.1007/s40520-020-01475-6)
Supplement: Supplementary file 8 — Supplementary file8 (PDF 479 kb) [file 40520_2020_1475_MOESM8_ESM.pdf]

Action Plans

Goals formulated by participants

In total, 22 participants allocated to the intervention group formulated 6 goals and 3 modifications each, comprising 132 goals and 66 modifications. The goals and environmental modifications fitted into three broad themes: modifying the physical environment, modifying movement patterns, and modifying the social environment (figure 1). Within modifying the environment, there were several common subthemes, namely: changing seating arrangements, using exercise equipment, modifying clothing, using reminders in the environment, and removing objects from around chairs. The theme of movement patterns was much broader, and encompassed goals around reducing many seated behaviours. These behaviours included TV viewing, computer/tablet/gaming device usage, drinking, eating and cooking, hobbies, reading, social activities, toileting, transport, and sitting in general. Within these themes were sub-themes for whether goals focused on breaking up sitting bouts, increasing standing, increasing sit-to-stand transitions, or increasing walking. Certain activities like TV viewing also contained goals that focused on reducing the incidence of that activity. Social modifications comprised of social reminders, encouragement, and co-opting tasks from others.

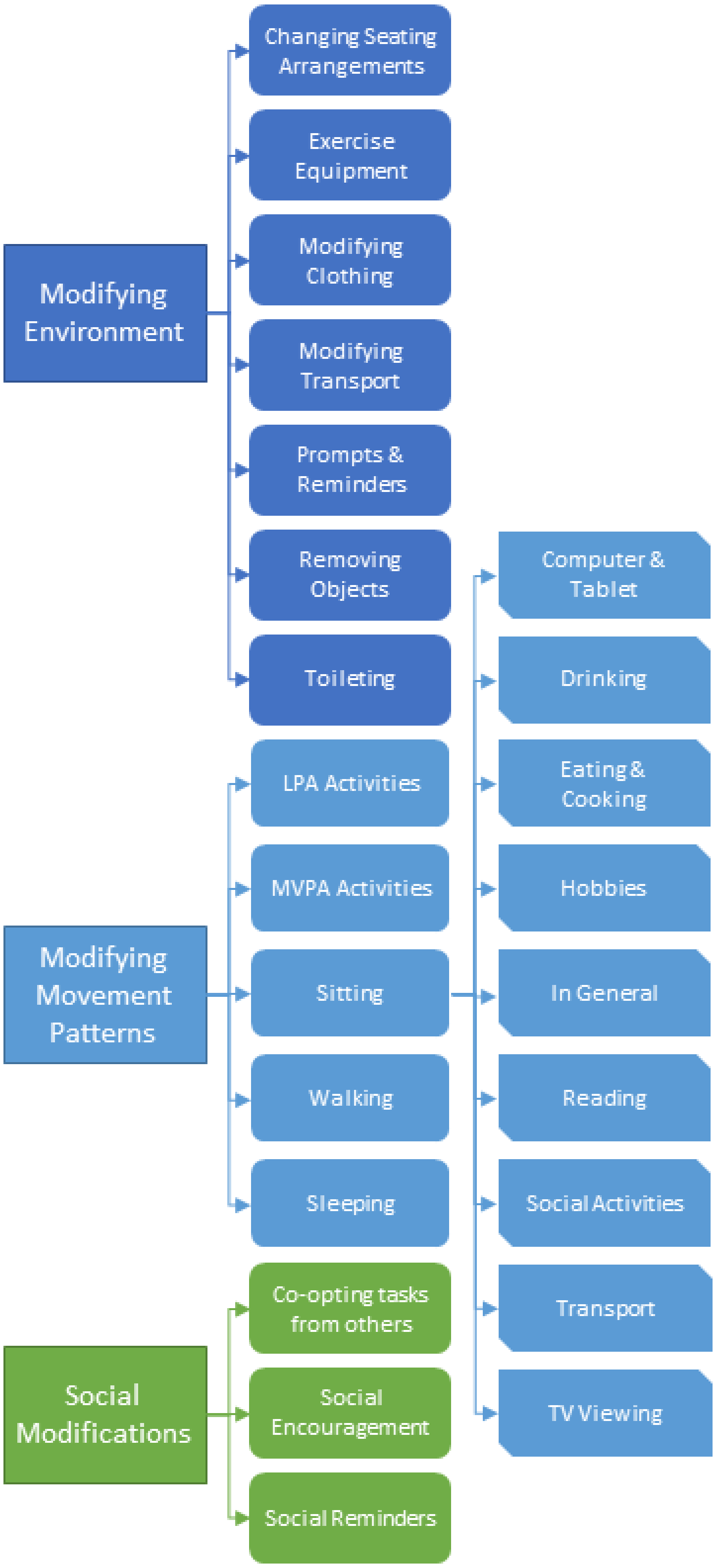

Figure 1. Higher-order coding categories for behavioural targets of goals.

Most of the goals related to sitting, with 71 goals relating to seated behaviours. After sitting, walking was assigned 44 codes, and LPA activities 11 codes. Within the theme of sitting, the most popular targeted behaviours were TV viewing (27 goals), eating and cooking (17), reading (10), and using the

computer or tablet (9). For walking, there was a relatively equal division between adding walks at various times of day (23) and modifying step targets (21). Goals relating to LPA activities primarily focused on increasing gardening (5) and household chores (5).

With respect to modifying the physical environment, the top themes were modifying clothing (e.g. wearing pedometer) (24), removing objects from vicinity of chairs (16), setting up environmental prompts (10), and modifying transportation (6). Social modifications encompassed co-opting tasks from others (3), social encouragement (2), and social reminders (2).

Overall, this coding of goals provided a comprehensive overview of the kinds of behaviours in which the participants were engaged, which were targeted to reduce their sedentariness.
